# An Efficient One-Step Reaction for the Preparation of Advanced Fused Bistetrazole-Based Primary Explosives

**DOI:** 10.1021/acscentsci.3c00219

**Published:** 2023-03-16

**Authors:** Wei Hu, Jie Tang, Xuehai Ju, Zhenxin Yi, Hongwei Yang, Chuan Xiao, Guangbin Cheng

**Affiliations:** † School of Chemistry and Chemical Engineering, Nanjing University of Science and Technology, Xiaolingwei 200, Nanjing 210094, China; ‡ China Northern Industries group Co., Ltd. (NORINCO GROUP), Beijing 100089, P. R. China

## Abstract

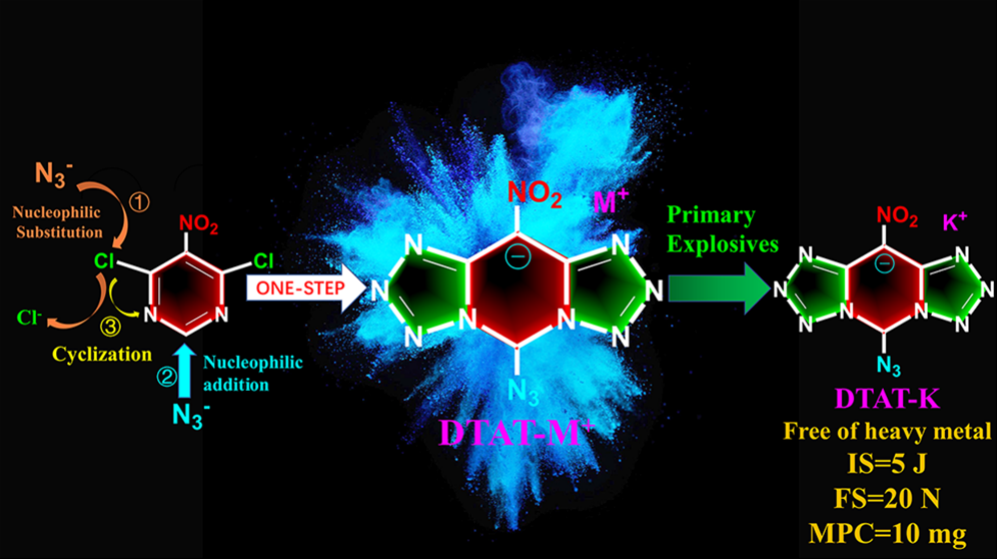

The first example of [5,6,5]-tricyclic bistetrazole-fused energetic materials has been obtained through a one-step reaction from commercial and inexpensive 4,6-dichloro-5-nitropyrimidine. This one-step reaction including nucleophilic substitution, nucleophilic addition, cyclization, and electron transfer is rarely reported, and the reaction mechanism and scope is well investigated. Among target compounds, organic salts exhibit higher detonation velocities (*D*: 8898–9077 m s^–1^) and lower sensitivities (IS: 16–20 J) than traditional high energy explosive RDX (*D* = 8795 m s^–1^; IS = 7.5 J). In addition, the potassium salt of 5-azido-10-nitro-bis(tetrazolo)[1,5-c:5′,1′-f]pyrimidin (**DTAT-K**) possesses excellent priming ability, comparable to traditional primary explosive Pb(N_3_)_2_, and ultralow minimum primary charge (MPC = 10 mg), which is the lowest MPC among the reported potassium-based primary explosives. The simple synthesis route, free of heavy metal and expensive raw materials, makes it promising to quickly realize this material in large-scale industrial production as a green primary explosive. This work accelerates the upgrade of green primary explosives and enriches future prospects for the design of energetic materials.

## Abstract 2

The first [5,6,5]-tricyclic bistetrazole-fused energetic materials have been synthesized in one-step reaction. **DTAT-K** has excellent priming ability, making it a promising green primary explosive.

With the development of economy and technology, the deep-exploration of natural resources has extended to extreme environments such as deep sea, deep well, and outer space.^1−3^ Therefore, the demands for energetic materials with higher energy, higher stability, and environmental protection is increasing.^4^ In the long term development of high energy density materials (HEDMs), thousands of HEDMs have been designed, but few ideal candidates have been achieved in practical application.^5^ Recently, nitrogen-rich heterocycles have become one of the most exciting scientific discoveries in the field of energetic materials.^6^ They often possess high energy with positive heats of formation due to a large number of N–N and C–N bonds.^7^ Most notably, the tetrazole backbone has become the focus of primary and secondary explosives owing to their high heats of formation and synthetic conveniences. Moreover, the heterocycle-fused backbone exhibits high energy and safety because of its tremendous ring strain energy and conjugated system.^8,9^

The design and synthesis of novel tetrazole-fused energetic materials are expected to satisfy the new generation of primary and secondary explosives. However, due to their poor stability and unavailable synthesis routes, no bistetrazole-fused compounds have been obtained. J. M. Shreeve et al. synthesized a tetrazole-fused primary explosive (**I**) with good detonation performance and ultralow minimum primary charge (MPC).^10^ Synthesis of the bistetrazole-fused compound **II** was also attempted, but failed (Figure 1). Thereafter, R. D. Gilardi et al. proposed the possibility of synthesis of a novel bistetrazole-fused structure (**IV**), but the subsequent work experimentally and theoretically disclosed that this structure was unstable and could easily decompose into **III**.^11−13^ Herein, we designed and synthesized several [5,6,5]-tricyclic bistetrazole-fused compounds through a one-step reaction from commercial and inexpensive 4,6-dichloro-5-nitropyrimidine.

**Figure 1:**
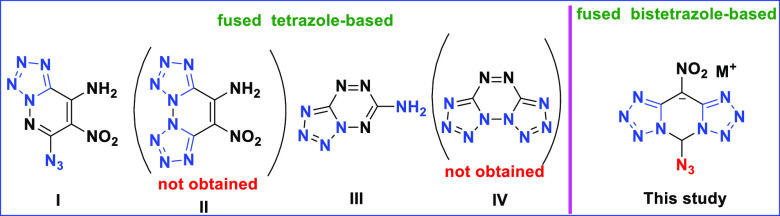
Structure and property of several tetrazole derivatives.

Generally, lead azide (LA) is the most widely used primary explosive with excellent priming ability. However, the serious heavy-metal pollution forced them to be replaced with a green explosive. To date, some representative potassium derivatives with environmental protection characteristics provide a promising platform for primary explosives such as K_2_DNABT, K_2_DNAT, and K_2_DNAAzT (Figure 2).^14−16^ However, the initiation ability of these potassium-based nitramino derivatives is weaker than that for LA. In addition, the extremely high sensitivity of these compounds to external stimuli makes safe handling in general, especially larger quantities, problematic, which limits their practical application. In view of the initiating unit of LA and environmental protection unit of potassium derivatives, we proposed a new primary explosive by introducing azido and potassium ion into the above-mentioned bistetrazole-fused backbone.

**Figure 2:**
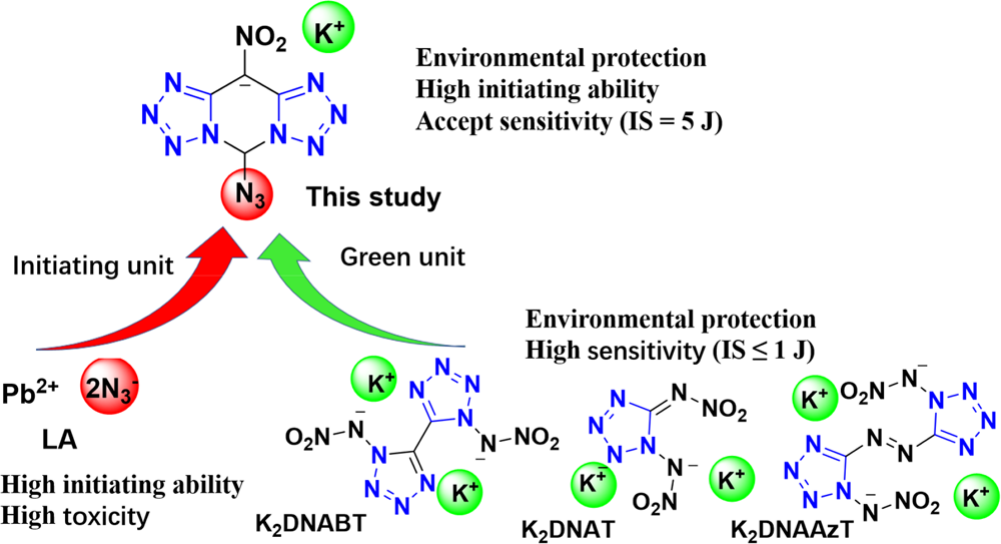
Structure and property of several primary explosives.

Initially, the substitution reaction of commercial and inexpensive 4,6-dichloro-5-nitropyrimidine (**1**) and NaN_3_ was attempted to afford 4,6-diazido-5-nitropyrimidine (**2**). Unexpectedly, a cyclization reaction occurred to form [5,6,5]-tricyclic bistetrazole-fused backbone instead of the expected substitution reaction (Scheme 1). To our surprise, another azido group was also introduced to the bistetrazole-fused backbone to form sodium 5-azido-10-nitro-bis(tetrazolo)[1,5-c:5′,1′-f]pyrimidinium (**3**). We also tried to obtain the neutral compound **3**-**2** by adjusting the pH of the aqueous solution of **3** with 20% H_2_SO_4_, 37% HCl, or 70% HClO_4_, respectively. However, the pyrimidine ring of compound **3** was broken to afford compound **3**-**1**, indicating the instability of the neutral compound of **3**. In order to increase the energy of [5,6,5]-tricyclic bistetrazole-fused compound, energetic salts **DTAT-K** and **5**-**7** were obtained through ion exchange of silver salt (**4**) and potassium chloride, ammonium chloride, hydrazinium chloride, and hydroxylammonium chloride, respectively.

**Scheme 1:**
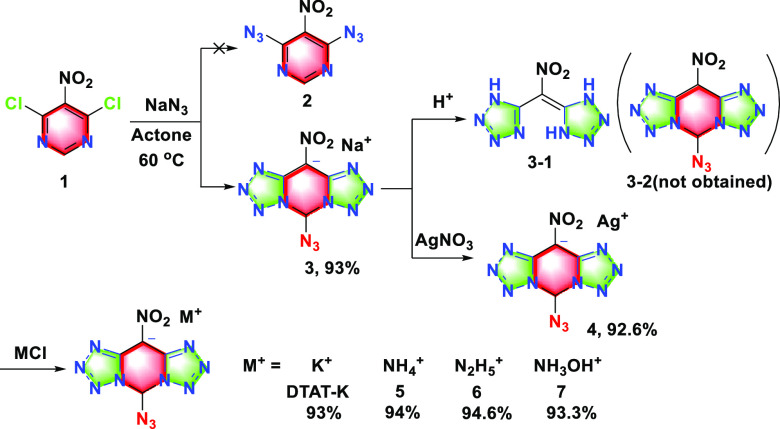
Synthetic Route of Fused Bistetrazole Derivatives

These results encouraged us to further investigate different 5-substituent 4,6-dichloro-pyrimidines to examine the scope of this one-step reaction system (Scheme 2). Several similar substrates (**A**: 5-bromo-4,6-dichloropyrimidine; **B**: 4,5,6-trichloropyrimidine; **C**: 4,6-dichloro-5-fluor opyrimidine) were tried under the same conditions (Scheme 2). The reaction of 5-substituent pyrimidine with Br (**A**) or Cl (**B**) groups with NaN_3_ only gave diazide-substituted products **A-1** and **B-1**, respectively. With the enhancement of a group’s electron-withdrawing ability of F, triazide-substituted product **C-1** was obtained. However, all these azides substituted products do not undergo the nucleophilic addition reaction of N_3_^–^ and the cyclization of two azide groups. It is noteworthy that the one-step reaction (substitution, cyclization, addition, and electron transfer) could only occur when the substitutional group was replaced by −NO_2_ with strong electron-withdrawing ability among four substrates. These interesting results show that this reaction could be related to the electron-withdrawing ability of the 5-substituent of pyrimidine.

**Scheme 2:**
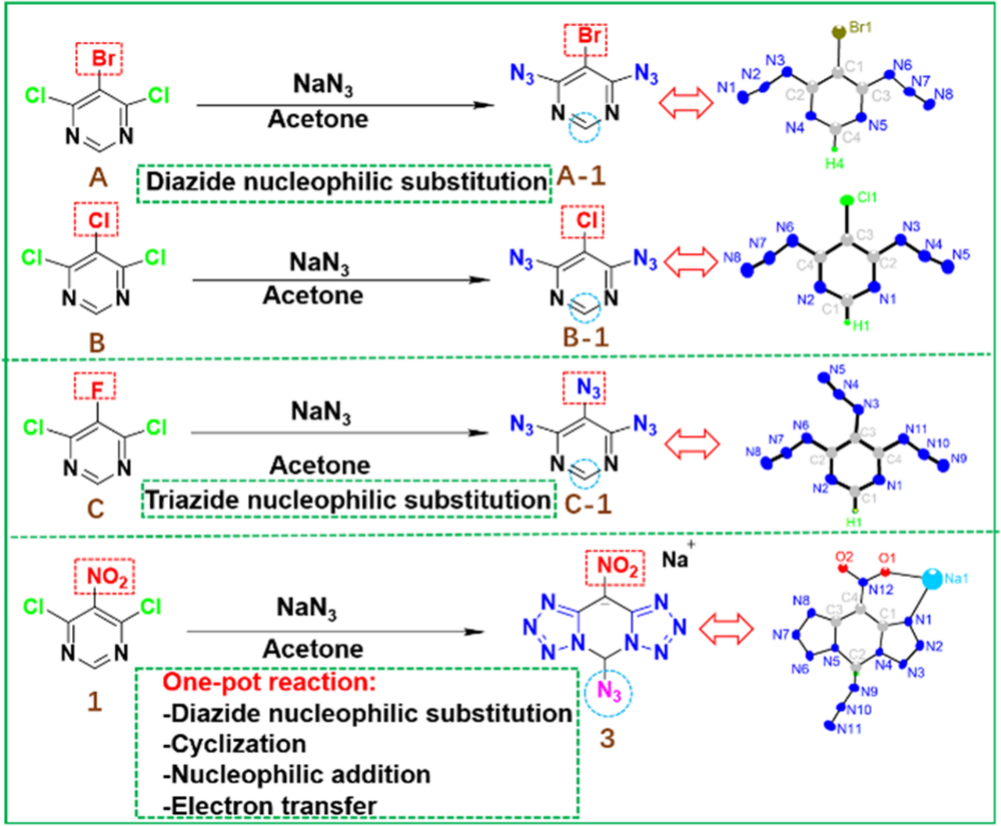
Reaction of Four Substrates with Sodium Azide

A possible reaction mechanism for the synthesis of **3** was illustrated in Scheme 3a, which could be supported by the reaction potential energy curves of the synthesis pathway (Figure 3) and Mulliken charges variations (Scheme 3b). As shown in Figure 3a, the chlorine atoms in **1** were replaced by azide anions in acetone to form intermediate **M1**, whose energy barrier was almost zero. Then two reaction processes were proposed as paths A (cyclization-addition) and B (addition-cyclization-electron transfer). In path A, azide groups in both the 1- and 5-positions attacked N atoms of pyrimidine in **M1** to form fused bistetrazole intermediate **M2**. Another azide anion attacked the C3 of pyrimidine in **M2** and combined with the sodium ion to form **3**. However, the relatively large reaction energy barrier of 87.17 kJ mol^–1^ and a cliff-like drop of the total energy (red line) shown in Figure 3c indicated that the pyrimidine ring in **M2** could be easily broken to form **M4** in the reaction process of **M2** with azide anions. So we turned our attention to investigating path B. First, the azide anion attacked the C3 of pyrimidine in **M1** to form intermediate **M4**. This process could be easily carried out due to the relatively low reaction energy barrier (10.88 kJ mol^–1^) in Figure 3b. The Mulliken charges variations of the C3 in **M4** (−0.716) was the lowest charge on the pyrimidine ring (Scheme 3b), indicating that the azide anion could be introduced into pyrimidine by the addition path. Then azide groups in both the 1- and 5-positions attacked the N atoms of pyrimidine in **M4** to form [5,6,5]-tricyclic bistetrazole-fuse backbone (Figure 3d). Meanwhile, the Mulliken charges variations changed from C3 (−0.716) and C6 (0.258) in **M4** to C3 (−0.186) and C6 (−0.344) in **3**, indicating that the negative charge could be transferred from C3 to C6 via a tetrazole ring. Finally, compound 3 was formed by combination with the sodium ion. Therefore, path B (addition-cyclization reaction) is reasonable for the one-step reaction.

**Scheme 3:**
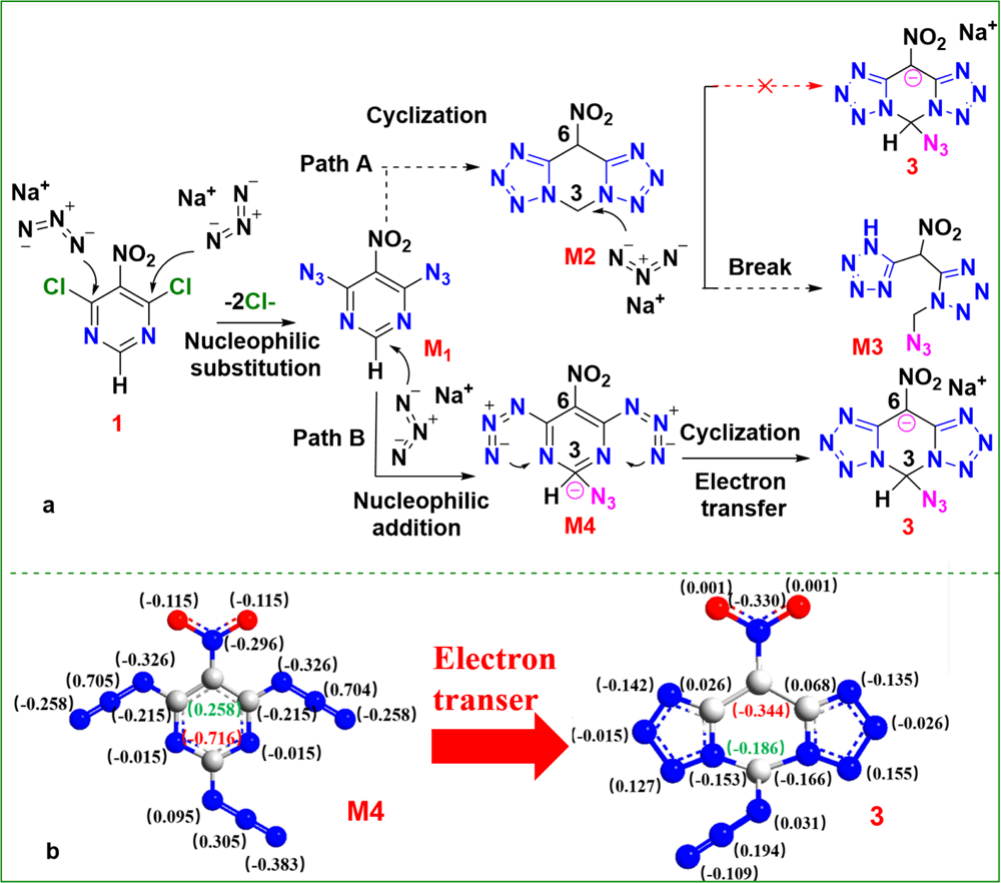
(a) The possible reaction process of compound **3**; (b) Mulliken charges variations of **M2** and compound **3**.

**Figure 3:**
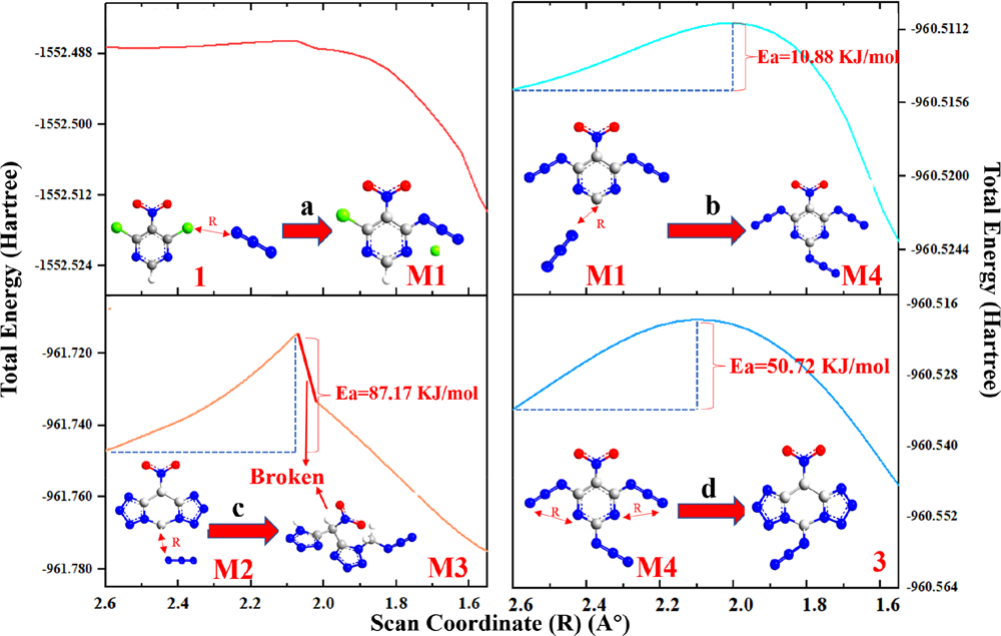
Energy profiles along the formation pathway of compound **3** at the B3LYP/6-311++G level. (**1** to **M1** for a; **M1** to **M4** for b; **M2** to **M3** for c; **M4** to **3** for d).

All samples must be dried in vacuum before growing the crystals to remove the solvent, as validated by DSC without any endothermic solvent peak (Figure S20). Crystals of target compounds **DTAT-K**, **5**, and **6** were obtained through evaporation of the water solution. Compound **5** crystallizes in triclinic space group *P*-1 with one **5** molecule and one water molecule in the unit cell (Figure 4a). The crystal density of **5**·H_2_O is 1.750 g cm^–3^ in 193 K. The torsion angles of N(1)–C(1)–C(2)–C(3) and N(11)–C(1)–C(2)–C(3) at 171.6(10)° in compound **5** are 171.6° and −169.0°, respectively, which indicating the tetrazole ring and pyrimidine ring are almost coplanar. The tetrazole ring and pyrimidine ring are almost coplanar, which is indicated by the torsion angles. As shown in Figure 4b, there are abundant intermolecular hydrogen bonds in compound **5**·H_2_O. These hydrogen bonds and the planar molecule make a face-to-face packing diagram of crystal **5**·H_2_O. The distance between the two layers of **5**·H_2_O is 3.73 Å, suggesting that two of the molecules are in the range of π–π interactions and packing tightly.^17^

**Figure 4:**
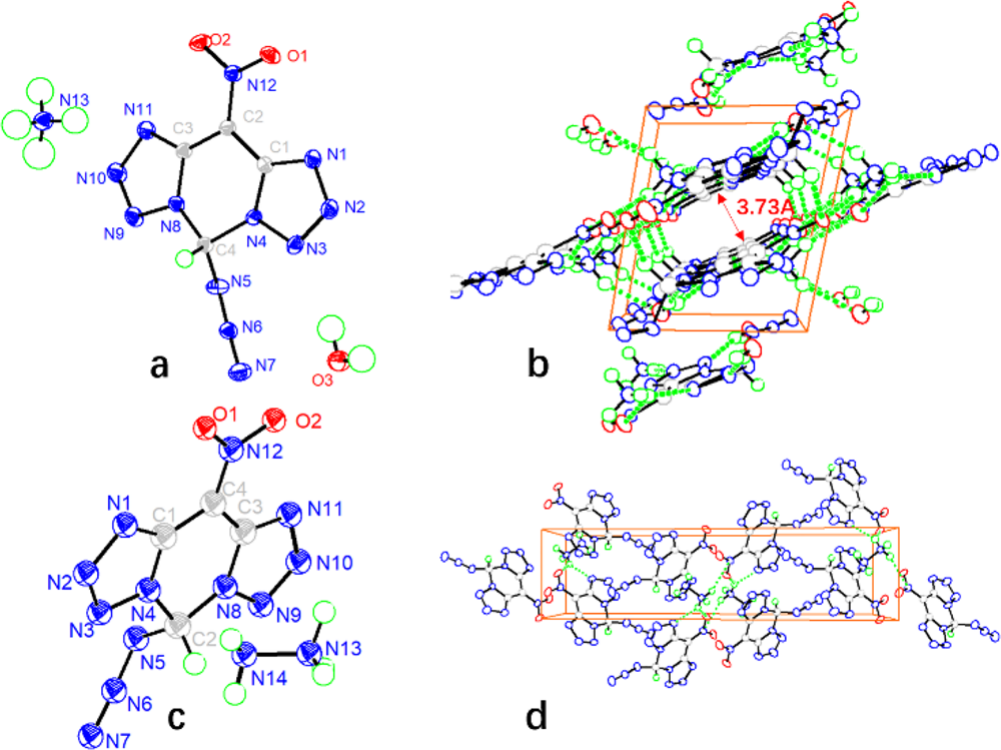
Single-crystal X-ray structures of **5**·H_2_O (a) and **6** (c); crystal packing diagram of **5**·H_2_O (b) and **6** (d) (green lines represent hydrogen bonds).

Compound **6** belongs to the monoclinic space group *C*2/*c* with four molecules per unit cell (*Z* = 4) and a crystal density of 1.774 g cm^–3^ at 193 K. Similar to compound **5**, the tetrazole ring, pyrimidine, and nitro group of compound **6** are coplanar, which is confirmed by the torsion angles of N(11)–C(3)–C(4)–N(12) and N(1)–C(1)–C(4)–N(12) at −2.8(4)° and 2.5(4)°. And the azido group is twisted out of the fused ring, which is demonstrated by the torsion angles of N(4)–C(2)–N(5)–N(6) (−99.2(3)°) and N(8)–C(2)–N(5)–N(6) (146.7(2)°) (Figure 4c). In addition, the inter- and intramolecular hydrogen bonds among the azido group, nitro group, pyrimidine ring, and hydrazine ion result in an edge-to-face arrangement of crystal **6** (Figure 4d).

**DTAT-K** crystallizes in the trigonal space group *R*3̅ with 18 formula units in the unit cell and a calculated density of 1.908 g cm^–3^ at 193 K. Each energetic ligand is connected to five potassium atoms via a K–O and K–N bonds (Figure 5a), and the bond length of K–O and K–N falls in the range of 2.734(3)–3.298(4) Å. The coordination environment results in the 3D crystal packing diagram of **DTAT-K** (Figure 5b). This packing diagram can also be viewed as a tubular structure. As shown in Figure 5c and 5d, the tubular framework structure is constructed through K–N and K–O bonds (polyhedrons centered on the potassium atom). The potassium atom, nitro group, and fused ring backbone give rise to tubular cavities, which can be viewed as a metal–organic framework (MOF). The sensitive azido groups are built into the cavity and thus can avoid direct triggers when external stimuli are acted upon. The tubular framework structure and hydrogen bonds (green lines in Figure 5b) enhance the thermal stability of **DTAT-K**.

**Figure 5:**
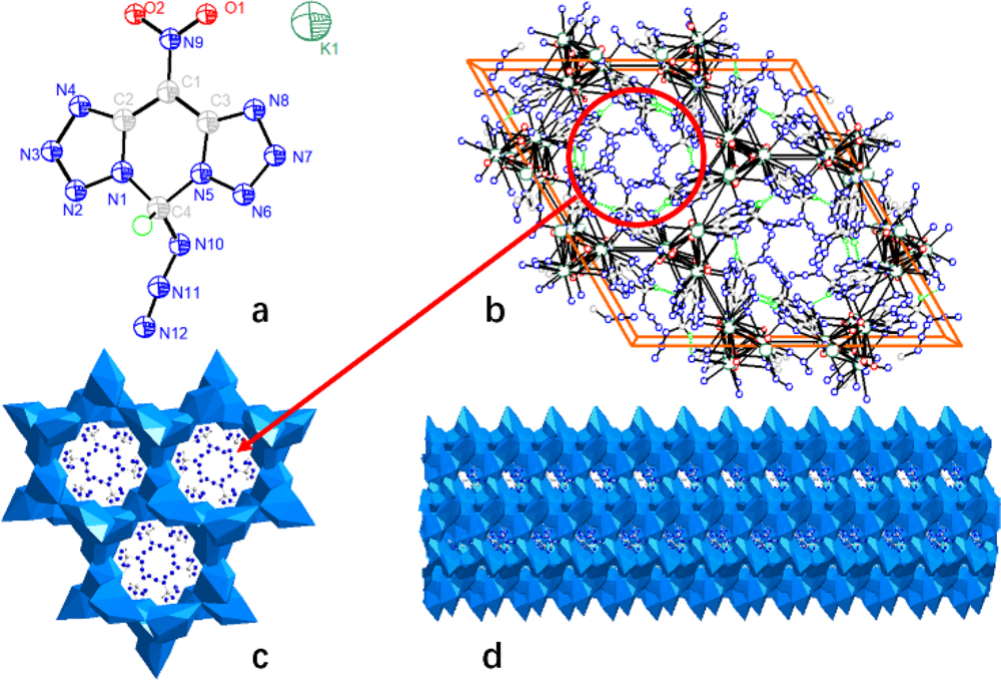
(a) Single-crystal X-ray structure; (b) crystal packing diagram of **DTAT-K** (green lines represent hydrogen bonds); (c) zeolitic MEP topology of **DTAT-K** (view along *c* axis); (d) zeolitic MEP topology of **DTAT-K** (view along *a* axis).

A series of tests, including density, thermal stability, sensitivity, and detonation performance, were employed to characterize the properties of target compounds. Those properties are shown in Table 1. The decomposition temperature of compounds is 135.3–163.6 °C. Among these compounds, **DTAT-K** possesses the highest thermal stability at 163.6 °C due to its crystal packing mode of the frame structure and hydrogen bonds, which is comparable to the scale-up and industrial applicable primary explosive DDNP (157 °C) (5,7-dinitrobenzo[d][1,2,3]oxadiazole).

**Table 1:** Properties of Target Compounds, DDNP, RDX, and Pb(N_3_)_2_

Compd.	*T*_d_^a^ [°C]	*P*^b^ [g cm^–3^]	△*H*_f_^c^ [kJ mol^–1^]/[kJ g^–1^]	*D*^d^ [m s^–1^]	*P*^e^ [GPa]	IS^f^ [J]	FS^g^[N]
**DTAT-K**	163.6	1.88	970.97/3.36	7917	25.2	5	20
**5**	135.3	1.77	1085.59/4.05	8898	31.4	18	240
**6**	154.5	1.76	1213.74/4.30	9077	31.9	16	160
**7**	139.6	1.78	1141.90/4.03	9044	33.1	20	240
DDNP^h^	157.0	1.72	327.0/1.56	6900	24.7	1	24.7
RDX^h^	204	1.80	70.3/0.36	8795	34.9	7.4	120
Pb(N_3_)_2_^h^	315	4.8	450.1/1.55	5877	33.4	0.6–4	0.3–0.5

a Decomposition temperature.

b Density determined by gas pycnometer at 25 °C.

c Calculated heat of formation.

d Detonation velocity.

e Detonation pressure.

f Impact sensitivity.

g Friction sensitivity.

h References (22−24).

The mechanical sensitivities of all compounds were determined by standard BAM methods.^18^ Organic salts **5**–**7** possess low impact sensitivities (IS > 16 J) and friction sensitivities (FS > 160 N), which are superior to those of RDX. It is notable that **DTAT-K** shows lower mechanical sensitivities (IS = 5 J) than Pb(N_3_)_2_ (IS = 0.6–4 J) and DDNP (IS = 1 J), which is consistent with crystal analysis, which guarantees the safety of production, handling, and transportation, and retains the reliable initiation capability of **DTAT-K**, simultaneously.

Generally, the heat of formation of energetic molecules can be increased by 364 kJ mol^–1^ through the introduction of an azido group.^19^ As a result, target compounds **5**–**7** and **DTAT-K** show high heat of formation in the range of 970.97 kJ mol^–1^ to 1213.74 kJ mol^–1^ (3.36 kJ g^–1^ to 4.30 kJ g^–1^). The densities of compounds were in the range of 1.77 g cm^–3^ to 1.88 g cm^–3^ which were measured by gas pycnometer at room temperature. Then the detonation velocities and pressures were calculated with EXPLO5 (version 6.05) based on their heats of formation and measured densities.^20^ As shown in Table 1, the detonation performance of **DTAT-K** is 7917 m s^–1^, which is much better than that of Pb(N_3_)_2_ (*D*: 5877 m s^–1^) and DDNP (*D*: 6900 m s^–1^). The organic salts **5**–**7** exhibit higher detonation velocities (*D*: 8898–9077 m s^–1^) than traditional high energy explosive RDX (*D*: 8795 m s^–1^). The high energy and low sensitivities of compounds **5**–**7** make them potential candidates for secondary energetic materials, especially for compound **6** (*D* = 9077 m s^–1^; *P* = 31.9 GPa; IS = 16 J; FS = 160 N).

The minimum priming charge (MPC) is the straightforward property to estimate the initiating abilities of primary explosives. To further assess the initiating ability of **DTAT-K**, the detonation test was performed, whose setup was shown in Figure 6a. The experimental work was performed by using a certain amount of **DTAT-K** to detonate 670 mg of secondary explosive (RDX) against a 5 mm lead block in comparison with the commonly used primary explosive Pb(N_3_)_2_. The apparatus was fired by an electric igniter. As shown in Figure 6b and 6c, the lead plate with a diameter of 40 mm was blown out of the hole (**DTAT-K**: φ = 24 mm; Pb(N_3_)_2_: φ = 23.5 mm) with 60 mg of primary explosive, indicating that the initiating ability of **DTAT-K** is comparable to Pb(N_3_)_2_. Then the charge amount of **DTAT-K** was reduced from 60 mg to 5 mg, and the MPC of **DTAT-K** is 10 mg according to PRC GJB 5891.19-2006,^21^ which is the lowest MPC among the reported potassium-based primary explosive (Figure 6d). The test results demonstrate that **DTAT-K** is an excellent green primary explosive and a promising replacement for Pb(N_3_)_2_.

**Figure 6:**
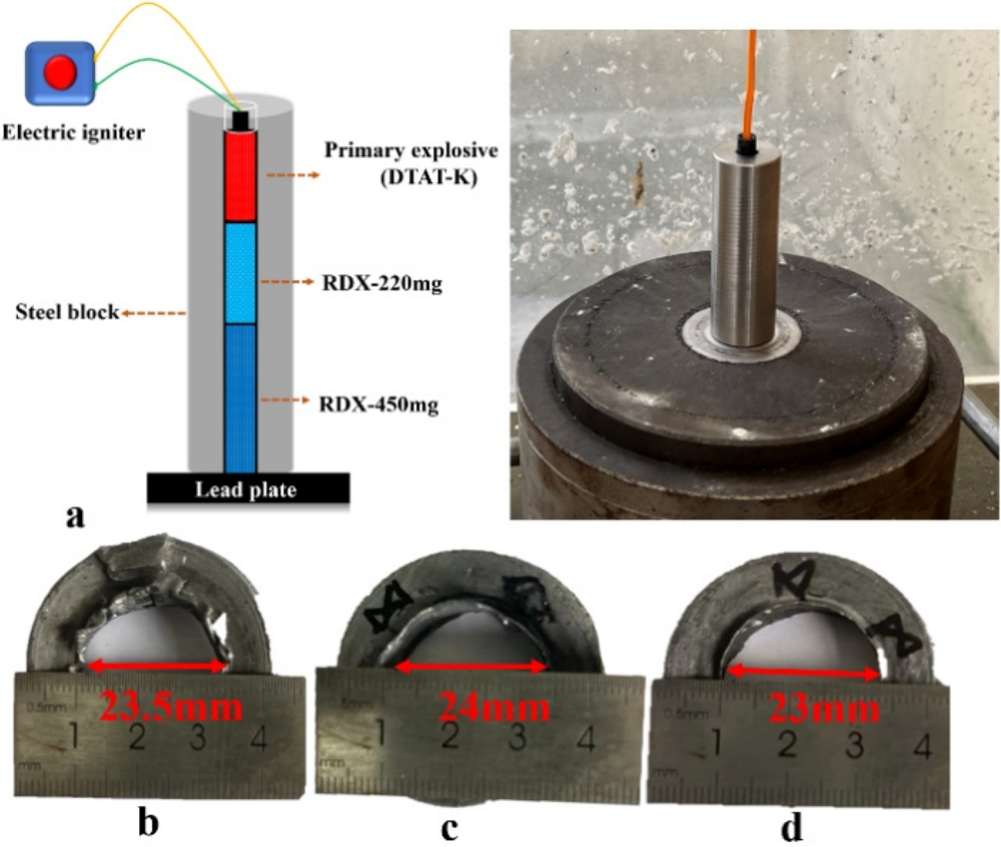
(a) Initiation capability test apparatus; (b) lead plates that were blasted out of the hole by 60 mg Pb(N_3_)_2_, (c) 60 mg **DTAT-K**, and (d)10 mg **DTAT-K**.

In conclusion, several [5,6,5]-tricyclic bistetrazole-fused energetic materials were first obtained through a one-step reaction from commercial and inexpensive 4,6-dichloro-5-nitropyrimidine. The structures of all target compounds were fully characterized by infrared spectroscopy (IR), multinuclear NMR (^1^H, ^13^C) spectroscopy, elemental analysis, and single-crystal X-ray diffraction. In the synthesis of energetic materials, the one-step process involving nucleophilic substitution, nucleophilic addition, cyclization, and electron transfer is rarely reported. This one-step reaction can only occur when the substitutional group was replaced by −NO_2_ with strong electron-withdrawing ability among four substrates. Furthermore, the proposed mechanism of this one-step reaction was proven by the potential energy curve. The desired results show that compounds **5**–**7** possess lower impact sensitivity (>16 J) and higher detonation velocities (8898–9077 m s^–1^) than traditional high-energy explosive RDX, making them potential candidates for secondary explosives. Moreover, the detonation test of **DTAT-K** demonstrates that the priming ability of **DTAT-K** is comparable to that of traditional primary explosives Pb(N_3_)_2_, while the energy and safety of **DTAT-K** is higher than that of Pb(N_3_)_2_. It is noteworthy that **DTAT-K** possesses excellent initiating ability and ultralow minimum priming charge (MPC = 10 mg), illustrating that **DTAT-K** may be used as an excellent green primary explosive and a promising replacement of Pb(N_3_)_2_. All those results demonstrated that this promising strategy would contribute to the expansion of exploration on new energetic materials from both academic and practical considerations.

## Supporting Information Available

Experimental Section, crystallographic data, NMR Spectra, DSC plots, IR curves, methodology and details for the heats of formation and other calculations (PDF)
Crystallographic file -P 2ac 2ab (CIF)
Crystallographic file -P 1 (CIF)
Crystallographic file -c 2yc (CIF)
Crystallographic file -R 3 (CIF)

## Supplementary Material

oc3c00219_si_003.pdf

oc3c00219_si_004.cif

oc3c00219_si_005.cif

oc3c00219_si_006.cif

oc3c00219_si_007.cif
