# Supplementary material for: An Efficient One-Step Reaction for the Preparation of Advanced Fused Bistetrazole-Based Primary Explosives
Source: ACS Cent Sci. 2023 Mar 16;9(4):742–7. doi: 10.1021/acscentsci.3c00219 (PMC10141573; doi:10.1021/acscentsci.3c00219)
Supplement: Supplementary file 1 — oc3c00219_si_003.pdf [file oc3c00219_si_003.pdf]

Supporting Information (SI)

An efficient one-step reaction for the preparation of advanced fused bistetrazole-based primary explosives

Wei Hu,[1†] Jie Tang,[1†] Xuehai Ju,[1] Zhenxin Yi,[1] Hongwei Yang,[1*] Chuan Xiao,[2*] Guangbin Cheng[1*]

1 School of Chemistry and Chemical Engineering, Nanjing University of Science and Technology, Xiaolingwei 200, Nanjing 210094, P. R. China.

2 China Northern Industries group Co., Ltd. (NORINCO GROUP), Beijing 100089, P. R. China

† These authors contributed equally to this work.

* Corresponding author;

*Email: gcheng@mail.njust.edu.cn (Guangbin Cheng)

*Email: 47785121@qq.com (Chuan Xiao)

*Email: hyang@mail.njust.edu.cn(Hongwei Yang)

`

Table of Contents

1. Experimental Section 3

2. The Crystallographic Data 4

3 Theoretical Study...14

4.Reference 16

5. NMR Spectras of Compounds 3-7 and DTAT-K...16

6. The TG-DSC Curves of 4-6 and DTAT-K...22

7. The IR curves of 4-6 and DTAT-K 23

`

1. Experimental Section

1.1. Safety Precaution

Although we experienced no difficulties in handling these energetic materials, small scale and best safety practices (leather gloves, face shield) are strongly encouraged. All chemical reagents, solvents were obtained by purchase and were used as supplied without further purification.

4,6-dichloro-5-nitropyrimidine can be obtained commercially.

1.2. Synthesis

1.2.1. Synthesis of DTAT-Na. (3)

Compound **1** (1.92 g, 10.0mmol) was dissolved in acetone (25 mL); then sodium azide (1.95 g, 30 mmol) was added. The reaction mixture was stirred at reflux for 12 h, then cooled to room temperature. The precipitate was filtered off and the filtrate was concentrated under reduced pressure to give **DTAT-Na**. (2.56 g, yield: 93%). ^{1}H NMR (300 MHz, DMSO-d6): δ = 9.35 (1H, s) ppm; ^{13}C NMR (125 MHz, DMSO-d6): δ = 148.5, 94.6, 79.4 ppm; IR (KBr): v = 3404, 2925, 2138, 1636, 1559, 1524, 1463, 1452, 1437, 1399, 1386, 1362, 1299, 1244, 1154, 1102, 1045, 978, 967, 872, 798, 755, 683, 550cm^{-1}; Elemental analysis calcd for $C_4HN_{12}O_2Na$ (272.12): C 17.66, H 0.37, N 61.77%; found: C 17.5, H 0.41, N 61.75%.

1.2.2 Synthesis of DTAT-Ag (4)

A solution of $AgNO_3$ (1.70 g ,10 mmol) in distilled water (10 mL) was added dropwise to the solution of ***DTAT-Na*** (2.72g, 10 mmol) in distilled water (15 mL) under stirring for 30 minutes. The precipitate was filtered, and thoroughly washed with distilled water (10 mL × 3) to obtain ***DTAT-Ag*** (3.3g, 92.6 %).

The solubility of DTAT-Ag is very poor, so there is no NMR spectrum; Elemental analysis for $C_4HN_{12}O_2Ag$ (357.00): calcd. C 13.46, H 0.28, N 47.08 %; found: C 13.28, H 0.30, N 47.09 %.

1.2.3 General procedure for synthesis of compounds DTAT-K, DTAT-NH$_4$ (5), DTAT- N$_2$H$_5$ (6), DTAT-NH$_3$OH (7)

Synthesis of DTAT-K

A solution of 0.45 mmol chloride salt in distilled water (10 mL) was added dropwise to the suspension of DTAT-Ag (0.16 mg, 0.45 mmol) in distilled water (15 mL). The mixture was stirred at 25 °C for 0.5 h, then the precipitate was filtered off and the filtrate was concentrated under reduced pressure to give the desired product.

DTAT-K： 0.12 mg (93.0%). ^{1}H NMR (500 MHz, d6-DMSO) δ 9.37(s). ^{13}C NMR (126 MHz, d6-DMSO) δ 148.5, 94.6, 79.4. IR (KBr pellet): ν 3404, 2925, 2138, 1636, 1559,1463, 1452, 1399, 1362, 1299, 1244, 1221, 1154, 1102, 1045, 978, 967, 872, 798, 755,683cm^{-1}; Elemental analysis for $C_4HN_{12}O_2$ (288.13): calcd. C 16.67, H 0.35, N 58.32 %; found: C 16.60, H 0.34, N 58.40 %.

DTAT-NH_4 (5)： 0.119 mg (94.0%). ^{1}H NMR (500 MHz, d6-DMSO) δ 9.35(s), 7.35 (s). ^{13}C NMR (126 MHz, d6-DMSO) δ148.5, 94.5, 79.4. IR (KBr pellet): ν 3138, 2901, 2114, 1566, 1526, 1463, 1451, 1435, 1409, 1387, 1360, 1299, 1255, 1210, 1188, 1133, 1097, 1041, 985, 954, 860, 798, 751, 729 cm^{-1}; Elemental analysis for $C_4H_5N_{13}O_2$(267.17): calcd. C 17.98, H 1.89, N 68.15 %; found: C 17.94, H 1.79, N 68.10 %.

DTAT-N_2H_5 (6)： 0.12 mg (94.6%). ^{1}H NMR (500 MHz, d6-DMSO) δ 9.34 (s). ^{13}C NMR (126 MHz, d6-DMSO) δ 148.5, 94.5, 79.4. IR (KBr pellet): ν 3361, 3281, 3168, 3042, 2136, 1590, 1560, 1526, 1478, 1463, 1433, 1392, 1325, 1302, 1248, 1221, 1130, 1099, 1079, 1034, 977, 952, 869, 748, 682cm^{-1}; Elemental analysis for $C_4H_6N_{14}O_2$ (282.19): calcd. C 17.03, H 2.14, N 69.49 %; found: C 17.10, H 2.12, N 69.51 %.

DTAT-NH_3OH (7)： 0.118 mg (93.3%). 1H NMR (500 MHz, d6-DMSO) δ 10.18 (s), 9.97 (s), 9.33 (s). 13C NMR (126 MHz, d6-DMSO) δ 148.5, 94.6, 79.5. IR (KBr pellet): ν 3138, 2920, 2674, 2136, 1607, 1561, 1532, 1463, 1435, 1408, 1386, 1341, 1302, 1255, 1229, 1210, 1187, 1160, 1117, 1094, 1037, 1004, 974, 922,873, 779, 749, 702cm^{-1}; Elemental analysis for $C_4H_5N_{13}O_3$ (283.17): calcd. C 16.97, H 1.78, N 64.30 %; found: C 16.90, H 1.79, N 64.40 %.

2. The Crystallographic Data

The crystal of **4, 5, 6** and **7** were performed on a Bruker Smart Apex II diffractometer with graphite-monochromated Mo Kα radiation (λ = 0.71073 Å), respectively. Integration and scaling of intensity data were accomplished using the SAINT program2. The structures were solved by intrinsic using SHELXT2014 and refinement was carried out by a full- matrix least-squares technique using SHELXT2014. The hydrogen atoms were refined isotropically, and the heavy atoms were refined anisotropically. N-H and O-H hydrogens were located from different electron density maps, and C-H hydrogens were placed in calculated positions and refined with a riding model. Data were corrected for the effects of absorption using SADABS4 Relevant crystal data and refinement results are summarized in Table S1

Table S1. Crystal data and structure refinement for **3, DTAT-K, 5** and **6.**

Crystal	**3**	**DTAT-K**	**5**	**6**
Chemical formula	$C_4H_7N_{12}NaO_5$	$C_4HKN_{12}O_2$	$C_4H_7N_{13}O_3$	$C_4H_6N_{14}O_2$
Formula weight	326.21	288.27	285.23	282.23
Temperature/K	193	193	193	193
Crystal system	orthorhombic	trigonal	triclinic	monoclinic
Space group	Pbca	R-3	P-1	C2/c
a/Å	16.3693(4)	24.7244(16)	6.6003(3)	13.9583(7)
b/Å	6.3669(2)	24.7244(16)	8.1646(4)	6.2279(4)
c/Å	22.4793(5)	8.5320(8)	10.6407(6)	24.6373(15)
α /°	90	90	72.718(2)	90
β /°	90	90	80.190(2)	99.268(2)
γ /°	90	120	77.721(2)	90
Volume/Å3	2342.83(11)	4516.8(7)	531.46(5)	2113.8(2)
Z	8	18	2	8
ρ calcg/cm3	1.85	1.908	1.782	1.774
μ /mm-1	1.742	0.557	0.152	0.147
F(000)	1328	2592	292	1152
Crystal size/mm3	0.15 × 0.12 × 0.11	0.15 × 0.13 × 0.12	0.13 × 0.12 × 0.1	0.15 × 0.13 × 0.12
Radiation	CuKα (λ = 1.54178)	MoKα (λ = 0.71073)	MoKα (λ = 0.71073)	MoKα (λ = 0.71073)
2Θ range for data collection/°	7.866 to 136.714	5.14 to 55.064	4.036 to 55.064	6.312 to 54.972
Index ranges	-19 ≤ h ≤ 19, -7 ≤ k ≤ 7, -27 ≤ l ≤ 27	-32 ≤ h ≤ 32, -31 ≤ k ≤ 26, -11 ≤ l ≤ 11	-8 ≤ h ≤ 8, -10 ≤ k ≤ 10, -13 ≤ l ≤ 13	-15 ≤ h ≤ 18, -8 ≤ k ≤ 8, -31 ≤ l ≤ 31
Reflections collected	44814	20707	17468	21630
Independent reflections	2148 [Rint = 0.0364, Rsigma = 0.0143]	2294 [Rint = 0.0587, Rsigma = 0.0303]	2431 [Rint = 0.0554, Rsigma = 0.0393]	2410 [Rint = 0.0956, Rsigma = 0.0472]

`

Data/restraints/parameters	2148/0/213	2294/0/172	2431/1/204	2410/0/201
Goodness-of-fit on F2	1.051	1.131	1.083	1.066
Final R indexes [I>=2σ (I)]	R1 = 0.0258, wR2 = 0.0715	R1 = 0.0782, wR2 = 0.1712	R1 = 0.0343, wR2 = 0.0903	R1 = 0.0555, wR2 = 0.1285
Final R indexes [all data]	R1 = 0.0275, wR2 = 0.0730	R1 = 0.0872, wR2 = 0.1756	R1 = 0.0367, wR2 = 0.0925	R1 = 0.0777, wR2 = 0.1449
Largest diff. peak/hole / e Å-3	0.23/-0.22	0.88/-0.51	0.30/-0.36	0.69/-0.38
CCDC number	2203196	2203197	2203198	2203199

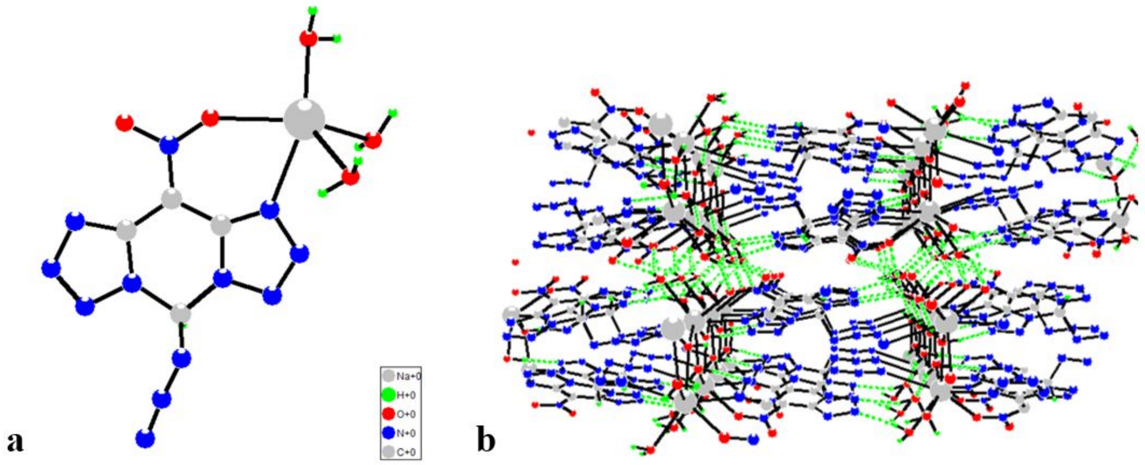

Figure S1 Single-crystal X-ray structures of **3**•3H_2O (a) and Crystal packing diagram of **3**•3H_2O (b)

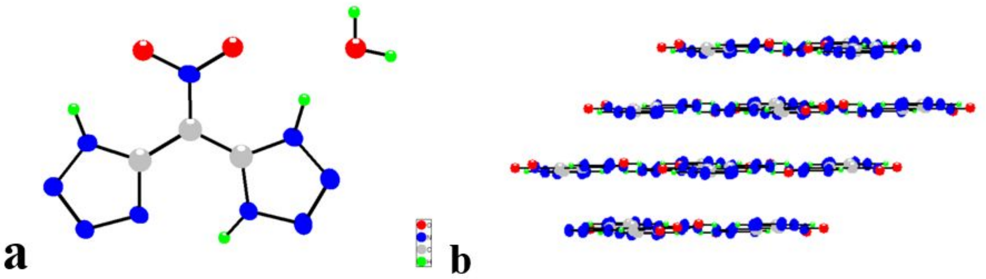

Figure S2 Single-crystal X-ray structures of **3-1**•H_2O (a) and Crystal packing diagram of **3-1**•H_2O (b)

`

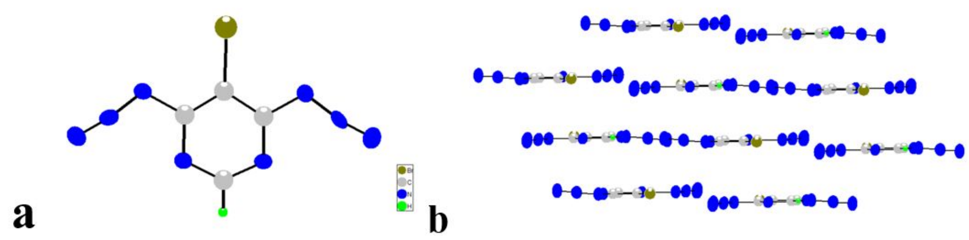

Figure S3 Single-crystal X-ray structures (a) and crystal packing diagram (b) of **A-1**

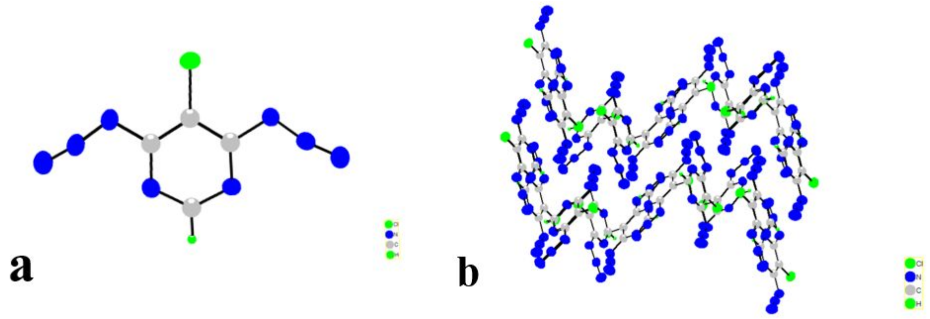

Figure S4 Single-crystal X-ray structures (a) and crystal packing diagram (b) of **B-1**

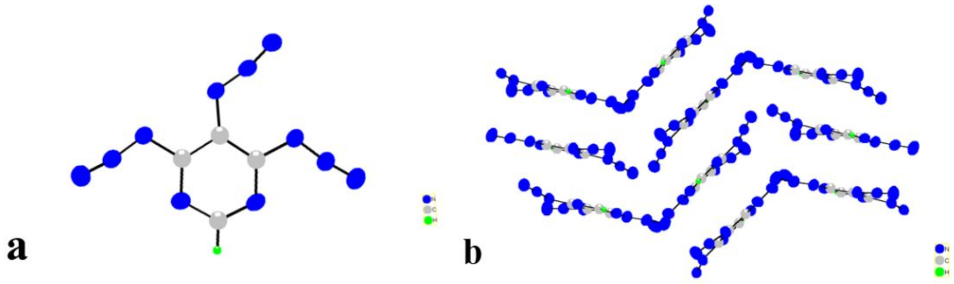

Figure S5 Single-crystal X-ray structures (a) and crystal packing diagram (b) of **C-1**

Table S2. Selected bond lengths [Å] and angles [°] for compound **DATA-K**

Parameter	Bond length (Å)	Parameter	Bond length (Å)
K(1)-K(1)^{1}	4.1096(12)	N(1)-C(4)	1.464(6)
K(1)-K(1)^{2}	4.1097(12)	N(2)-N(3)	1.279(6)
K(1)-O(1)	2.734(3)	N(3)-N(4)	1.367(5)
K(1)-O(1)^{1}	2.943(3)	N(4)-C(2)	1.326(5)
K(1)-O(2)^{3}	2.767(4)	N(5)-N(6)	1.362(5)
K(1)-O(2)^{1}	2.853(3)	N(5)-C(3)	1.356(6)
K(1)-N(3)^{4}	3.298(4)	N(5)-C(4)	1.474(6)
K(1)-N(4)^{3}	3.052(4)	N(6)-N(7)	1.299(7)

K(1)-N(4)[4]	3.007(4)	N(7)-N(8)	1.366(6)
K(1)-N(7)[5]	2.839(4)	N(8)-C(3)	1.329(5)
K(1)-N(8)	2.926(4)	N(9)-C(1)	1.355(6)
K(1)-N(9)[1]	3.260(4)	N(10)-N(11)	1.215(7)
O(1)-N(9)	1.264(5)	N(10)-C(4)	1.435(7)
O(2)-N(9)	1.263(5)	N(11)-N(12)	1.090(8)
N(1)-N(2)	1.355(5)	C(1)-C(2)	1.430(6)
N(1)-C(2)	1.356(6)	C(1)-C(3)	1.438(6)

[1]1/3+Y-X,5/3-X,-1/3+Z; [2]5/3-Y,4/3+X-Y,1/3+Z; [3]5/3-Y,4/3+X-Y,-2/3+Z; [4]+X,+Y,-1+Z; [5]4/3-X,5/3-Y,-1/3-Z

Parameter	Bond angle (°)	Parameter	Bond angle (°)
K(1)1-K(1)-K(1)[2]	102.61(3)	N(8)-K(1)-O(1)1	110.76(11)
O(1)-K(1)-K(1)[1]	144.77(8)	N(8)-K(1)-N(3)4	74.94(11)
O(1)-K(1)-K(1)[2]	45.68(7)	N(8)-K(1)-N(4)4	96.59(11)
O(1)1-K(1)-K(1)[2]	81.74(6)	N(8)-K(1)-N(4)3	122.09(11)
O(1)1-K(1)-K(1)[1]	41.65(6)	N(8)-K(1)-N(9)1	110.02(10)
O(1)-K(1)-O(1)[1]	108.29(10)	N(9)1-K(1)-K(1)1	58.06(6)
O(1)-K(1)-O(2)[3]	114.12(10)	N(9)1-K(1)-K(1)2	59.26(6)
O(1)-K(1)-O(2)[1]	65.56(9)	N(9)1-K(1)-N(3)4	87.76(10)
O(1)1-K(1)-N(3)[4]	66.92(10)	K(1)-O(1)-K(1)2	92.68(10)
O(1)-K(1)-N(3)[4]	129.46(10)	N(9)-O(1)-K(1)	128.1(3)
O(1)-K(1)-N(4)[4]	153.90(11)	N(9)-O(1)-K(1)2	92.9(2)
O(1)1-K(1)-N(4)[4]	68.18(10)	K(1)6-O(2)-K(1)2	93.96(10)
O(1)-K(1)-N(4)[3]	70.18(10)	N(9)-O(2)-K(1)2	97.1(2)
O(1)1-K(1)-N(4)[3]	110.71(10)	N(9)-O(2)-K(1)6	130.9(3)
O(1)-K(1)-N(7)[5]	112.24(12)	N(2)-N(1)-C(2)	109.5(4)
O(1)-K(1)-N(8)	59.55(10)	N(2)-N(1)-C(4)	119.0(4)
O(1)1-K(1)-N(9)[1]	22.78(8)	C(2)-N(1)-C(4)	131.4(4)
O(1)-K(1)-N(9)[1]	88.15(9)	N(3)-N(2)-N(1)	105.3(4)
O(2)3-K(1)-K(1)[1]	43.84(7)	N(2)-N(3)-K(1)7	145.5(3)
O(2)1-K(1)-K(1)[2]	42.19(7)	N(2)-N(3)-N(4)	112.5(4)
O(2)3-K(1)-K(1)[2]	69.22(7)	N(4)-N(3)-K(1)7	65.8(2)
O(2)1-K(1)-K(1)[1]	80.22(7)	K(1)7-N(4)-K(1)6	85.41(10)
O(2)3-K(1)-O(1)[1]	63.93(9)	N(3)-N(4)-K(1)7	89.7(2)
O(2)1-K(1)-O(1)[1]	44.21(9)	N(3)-N(4)-K(1)6	125.4(3)
O(2)3-K(1)-O(2)[1]	73.78(3)	C(2)-N(4)-K(1)6	112.4(3)

O(2)3-K(1)-N(3)^{4}	107.96(10)	C(2)-N(4)-K(1)7	140.3(3)
O(2)1-K(1)-N(3)^{4}	102.63(11)	C(2)-N(4)-N(3)	105.5(4)
O(2)1-K(1)-N(4)^{4}	111.47(10)	N(6)-N(5)-C(4)	119.7(4)
O(2)1-K(1)-N(4)^{3}	85.76(10)	C(3)-N(5)-N(6)	109.4(4)
O(2)3-K(1)-N(4)^{3}	56.90(10)	C(3)-N(5)-C(4)	130.9(4)
O(2)3-K(1)-N(4)^{4}	88.17(10)	N(7)-N(6)-N(5)	104.9(4)
O(2)3-K(1)-N(7)^{5}	91.24(12)	N(6)-N(7)-K(1)5	128.1(3)
O(2)1-K(1)-N(8)	97.31(10)	N(6)-N(7)-N(8)	112.3(4)
O(2)3-K(1)-N(8)	170.99(10)	N(8)-N(7)-K(1)5	107.3(3)
O(2)3-K(1)-N(9)^{1}	62.10(9)	N(7)-N(8)-K(1)	127.0(3)
O(2)1-K(1)-N(9)^{1}	22.60(9)	C(3)-N(8)-K(1)	118.7(3)
N(3)4-K(1)-K(1)^{2}	144.78(8)	C(3)-N(8)-N(7)	105.7(4)
N(3)4-K(1)-K(1)^{1}	64.32(8)	O(1)-N(9)-K(1)2	64.4(2)
N(4)4-K(1)-K(1)^{1}	47.74(8)	O(1)-N(9)-C(1)	120.9(4)
N(4)4-K(1)-K(1)^{2}	148.34(8)	O(2)-N(9)-K(1)2	60.3(2)
N(4)3-K(1)-K(1)^{1}	100.38(7)	O(2)-N(9)-O(1)	119.5(4)
N(4)3-K(1)-K(1)^{2}	46.84(7)	O(2)-N(9)-C(1)	119.5(3)
N(4)3-K(1)-N(3)^{4}	160.35(10)	C(1)-N(9)-K(1)2	158.2(3)
N(4)4-K(1)-N(3)^{4}	24.49(10)	N(11)-N(10)-C(4)	112.4(5)
N(4)4-K(1)-N(4)^{3}	135.86(12)	N(12)-N(11)-N(10)	168.8(7)
N(4)4-K(1)-N(9)^{1}	90.95(10)	N(9)-C(1)-C(2)	122.1(4)
N(4)3-K(1)-N(9)^{1}	94.32(10)	N(9)-C(1)-C(3)	121.9(4)
N(7)5-K(1)-K(1)^{2}	121.77(11)	C(2)-C(1)-C(3)	115.9(4)
N(7)5-K(1)-K(1)^{1}	97.26(10)	N(1)-C(2)-C(1)	119.4(4)
N(7)5-K(1)-O(1)^{1}	138.52(12)	N(4)-C(2)-N(1)	107.3(4)
N(7)5-K(1)-O(2)^{1}	160.97(13)	N(4)-C(2)-C(1)	133.3(4)
N(7)5-K(1)-N(3)^{4}	93.02(12)	N(5)-C(3)-C(1)	119.3(4)
N(7)5-K(1)-N(4)^{3}	76.09(12)	N(8)-C(3)-N(5)	107.7(4)
N(7)5-K(1)-N(4)^{4}	78.97(12)	N(8)-C(3)-C(1)	133.0(4)
N(7)5-K(1)-N(8)	97.17(13)	N(1)-C(4)-N(5)	103.2(4)
N(7)5-K(1)-N(9)^{1}	151.97(12)	N(10)-C(4)-N(1)	111.9(4)
N(8)-K(1)-K(1)1	137.28(9)	N(10)-C(4)-N(5)	106.1(4)
N(8)-K(1)-K(1)2	103.34(8)		

^{1}1/3+Y-X,5/3-X,-1/3+Z; ^{2}5/3-Y,4/3+X-Y,1/3+Z; ^{3}5/3-Y,4/3+X-Y,-2/3+Z; ^{4}+X,+Y,-1+Z; ^{5}4/3-X,5/3-Y,-1/3-Z; ^{6}1/3+Y-X,5/3-X,2/3+Z; ^{7}+X,+Y,1+Z

Table S3. Hydrogen bonds present in compound **DATA-K**

D-H•••A	d(D-H)/ Å	d(H•••A)/ Å	d(D•••A)/ Å	<(DHA)/ °
N(7) -H(7A) •••O(9)	0.88	2.22	2.989(7)	146.0
O(3) -H(3A) •••N(10)	0.87	2.11	2.9720(14)	172
O(3) -H(3B) •••N(3)	0.85(2)	2.32(2）	3.1613(13)	170.7(19)
N(13) -H(13A) •••O(2)	0.936(19)	2.462(19)	2.9676(14)	113.9(15)
N(13) -H(13A) •••N(11)	0.936(19)	2.03(2)	2.9485(14)	168.6(15)
N(13) -H(13B) •••O(1)	0.95(2)	2.45(2)	3.0905(13)	124.7(16)
N(13) - H(13B) •••O(2)	0.95(2)	2.08(2)	2.9975(15)	164(2)
N(13) -H(13B) •••N(12)	0.95(2)	2.60(2)	3.4550(15)	150.3(18)
N(13) -H(13C) •••N(2)	0.89(2)	2.220(19)	3.1078(14)	171.8(18)
N(13)-H(13D)•••O(3)	0.88(2)	2.11(2)	2.9454(15)	158(2)
C(4)-H(4)•••O(1)	1	2.46	3.2198(14)	132

Table S4. Selected bond lengths [Å] and angles [°] for compound **5**

Parameter	Bond length (Å)	Parameter	Bond length (Å)
O1-N12	1.2555(12)	N8-N9	1.3547(14)
O2-N12	1.2572(12)	N8-C3	1.3570(14)
O3-H3A	0.87	N9-N10	1.2823(14)
O3-H3B	0.85(2)	N10-N11	1.3679(15)
N1-N2	1.3604(13)	N11-C3	1.3307(14)
N1-C1	1.3341(14)	N12-C2	1.3679(14)
N2-N3	1.2891(14)	N13-H13A	0.936(19)
N3-N4	1.3552(12)	N13-H13C	0.89(2)
N4-C4	1.4668(14)	N13-H13D	0.88(2)
N4-C1	1.3546(13)	N13-H13B	0.95(2)
N5-C4	1.4524(14)	C1-C2	1.4273(14)
N5-N6	1.2546(13)	C2-C3	1.4304(15)
N6-N7	1.1193(14)	C4-H4	1
N8-C4	1.4521(14)		

Parameter	Bond angle (°)	Parameter	Bond angle (°)
H3A-O3-H3B	104	H13A-N13-H13D	113.3(17)
N2-N1-C1	105.34(9)	H13B-N13-H13C	111.4(17)
N1-N2-N3	112.39(9)	H13B-N13-H13D	99(2)
N2-N3-N4	105.55(9)	H13C-N13-H13D	111.9(18)
N3-N4-C4	121.14(8)	N4-C1-C2	119.26(9)

`

C1-N4-C4	129.71(8)	N1-C1-N4	107.70(9)
N3-N4-C1	109.02(9)	N1-C1-C2	133.03(10)
N6-N5-C4	113.00(9)	C1-C2-C3	115.57(9)
N5-N6-N7	70.73(11)	N12-C2-C1	122.40(10)
N9-N8-C3	109.32(9)	N12-C2-C3	122.02(9)
N9-N8-C4	121.25(9)	N8-C3-C2	119.81(9)
C3-N8-C4	129.41(9)	N8-C3-N11	107.28(9)
N8-N9-N10	105.61(9)	N11-C3-C2	132.83(10)
N9-N10-N11	112.22(9)	N4-C4-N5	111.28(8)
N10-N11-C3	105.57(9)	N4-C4-N8	104.06(8)
O1-N12-C2	119.66(9)	N5-C4-N8	106.44(8)
O2-N12-C2	119.31(9)	N4-C4-H4	112
O1-N12-O2	121.02(9)	N5-C4-H4	112
H13A-N13-H13B	111.2(17)	N8-C4-H4	112
H13A-N13-H13C	109.9(17)		

Table S5. Hydrogen bonds present in compound **5**

D-H•••A	d(D-H)/ Å	d(H•••A)/ Å	d(D•••A)/ Å	<(DHA)/ °
O3-H3A•••N10	0.87	2.11	2.9720(14)	172
O3-H3B•••N3	0.85(2)	2.32(2)	3.1613(13)	170.7(19)
N13-H13A•••O2	0.936(19)	2.462(19)	2.9676(14)	113.9(15)
N13-H13A•••N11	0.936(19)	2.03(2)	2.9485(14)	168.6(15)
N13-H13B•••O1	0.95(2)	2.45(2)	3.0905(13)	124.7(16)
N13-H13B•••O2	0.95(2)	2.08(2)	2.9975(15)	164(2)
N13-H13B•••N12	0.95(2)	2.60(2)	3.4550(15)	150.3(18)
N13-H13C•••N2	0.89(2)	2.220(19)	3.1078(14)	171.8(18)
N13-H13D•••O3	0.88(2)	2.11(2)	2.9454(15)	158(2)
C4-H4•••O1	1	2.46	3.2198(14)	132

Table S6. Selected bond lengths [Å] and angles [°] for compound **3**

parameter	bond lengths/ Å	parameter	angles/ °
O1-N12	1.2601(14)	N5 -N6	1.3513(14)
O2-N12	1.2583(14)	N5 -C3	1.3530(16)
O3-H3A	0.83(2)	N5 -C2	1.4644(16)
O3-H3B	0.81(2)	N6 -N7	1.2905(16)
O4-H4B	0.88	N7 -N8	1.3630(16)
O4-H4A	0.88	N8 -C3	1.3350(17)

O5-H5A	0.87	N9 -N10	1.2534(16)
O5-H5B	0.87	N9 -C2	1.4568(17)
N1-N2	1.3668(17)	N10-N11	1.1222(17)
N1-C1	1.3274(16)	N12-C4	1.3629(17)
N2-N3	1.2841(16)	C1 -C4	1.4335(17)
N3-N4	1.3550(15)	C3 -C4	1.4286(19)
N4-C2	1.4497(16)	C2 -H2	0.989(15)
N4-C1	1.3570(16)		

Parameter	Bond angle (°)	Parameter	Bond angle (°)
H3A-O3 -H3B	108(2)	O1 -N12-C4	119.96(11)
H4A-O4 -H4B	104	O2 -N12-C4	119.75(11)
H5A-O5 -H5B	105	N4 -C1 -C4	119.77(11)
N2 -N1 -C1	105.45(10)	N1 -C1 -N4	107.53(11)
N1 -N2 -N3	112.36(11)	N1 -C1 -C4	132.67(12)
N2 -N3 -N4	105.44(10)	N4 -C2 -N9	107.81(10)
C1 -N4 -C2	129.16(10)	N4 -C2 -N5	104.18(9)
N3 -N4 -C1	109.22(10)	N5 -C2 -N9	110.38(10)
N3 -N4 -C2	121.12(10)	N5 -C3 -N8	107.33(11)
N6 -N5 -C2	120.29(9)	N5 -C3 -C4	119.08(11)
N6 -N5 -C3	109.56(10)	N8 -C3 -C4	133.59(12)
C2 -N5 -C3	130.07(10)	C1 -C4 -C3	115.68(11)
N5 -N6 -N7	105.41(10)	N12-C4 -C1	122.19(12)
N6 -N7 -N8	112.21(11)	N12-C4 -C3	121.96(11)
N7 -N8 -C3	105.49(11)	N4 -C2 -H2	111.1(9)
N10-N9 -C2	112.48(11)	N5 -C2 -H2	109.3(8)
N9 -N10-N11	171.28(14)	N9 -C2 -H2	113.7(9)
O1 -N12-O2	120.28(11)		

Table S7. Hydrogen bonds present in compound **3**

D-H•••A	d(D-H)/ Å	d(H•••A)/ Å	d(D•••A)/ Å	<(DHA)/ °
O3- H3A•••N2	0.83(2)	2.23(2)	3.0030(16)	154.1(18)
O3-H3B•••O5	0.81(2)	2.03(3)	2.8007(15)	157(2)
O4-H4A •••O1	0.88	2.37	3.1737(15)	151
O4-H4A•••O2	0.88	2.23	3.0075(13)	147
O4-H4B •••N7	0.88	2.19	3.0370(15)	163
O5-H5A•••O4	0.87	1.97	2.8168(14)	163
O5-H5B •••O1	0.87	2.53	3.0525(14)	119

O5-H5B•••N6	0.87	2.37	3.1126(16)	144
C2-H2•••N8	0.989(15)	2.386(13)	3.2140(17)	140.9(12)
C2-H2 •••O2	0.989(15)	2.448(16)	3.1974(16)	132.2(11)

Table S8. Selected bond lengths [Å] and angles [°] for compound **6**

parameter	bond lengths/ Å	parameter	angles/ °
O1-N12	1.272(3)	N9-N10	1.285(3)
O2-N12	1.260(3)	N10-N11	1.372(3)
N1-N2	1.369(3)	N11-C3	1.326(3)
N1-C1	1.323(3)	N12-C4	1.357(3)
N2-N3	1.283(4)	N13-N14	1.435(3)
N3-N4	1.357(3)	N13-H13A	0.90(3)
N4-C1	1.349(3)	N13-H13B	0.97(4)
N4-C2	1.454(3)	N13-H13C	0.89(4)
N5-N6	1.250(3)	N14-H14B	0.84(4)
N5-C2	1.440(3)	N14-H14A	0.90(4)
N6-N7	1.126(4)	C1-C4	1.430(3)
N8-N9	1.356(3)	C3-C4	1.433(3)
N8-C2	1.453(3)	C2-H2	
N8-C3	1.355(3)		

Parameter	Bond angle (°)	Parameter	Bond angle (°)
N2-N1-C1	105.9(2)	H13A-N13-H13C	111(3)
N1-N2-N3	111.8(2)	H13B-N13-H13C	107(3)
N2-N3-N4	105.5(2)	N13-N14-H14A	105(2)
N3-N4-C1	109.4(2)	N13-N14-H14B	108(2)
N3-N4-C2	119.9(2)	H14A-N14-H14B	108(4)
C1-N4-C2	130.5(2)	N1-C1-C4	133.1(2)
N6-N5-C2	111.9(2)	N4-C1-C4	119.5(2)
N5-N6-N7	171.4(3)	N1-C1-N4	107.4(2)
N9-N8-C2	120.78(19)	N4-C2-N5	110.6(2)
N9-N8-C3	109.3(2)	N5-C2-N8	108.7(2)
C2-N8-C3	129.9(2)	N4-C2-N8	104.45(19)
N8-N9-N10	105.4(2)	N8-C3-N11	107.7(2)
N9-N10-N11	112.2(2)	N11-C3-C4	132.5(2)
N10-N11-C3	105.36(19)	N8-C3-C4	119.8(2)
O1-N12-O2	120.11(19)	N12-C4-C3	122.2(2)
O1-N12-C4	119.14(19)	C1-C4-C3	115.4(2)
O2-N12-C4	120.75(19)	N12-C4-C1	122.4(2)

`

N14-N13-H13A	107(2)	N8-C2-H2	111
N14-N13-H13B	111(2)	N4-C2-H2	111
N14-N13-H13C	112(2)	N5-C2-H2	111
H13A-N13-H13B	108(3)	--	

Table S9. Hydrogen bonds present in compound **6**

D-H•••A	d(D-H)/ Å	d(H•••A)/ Å	d(D•••A)/ Å	<(DHA)/ °
N13-H13A•••O1	0.90(3)	2.24(3)	2.908(3)	131(3)
N13-H13A•••N1	0.90(3)	2.10(3)	2.870(3)	143(3)
N13-H13B•••O1	0.97(4)	1.91(4)	2.868(3)	169(3)
N13-H13B•••N12	0.97(4)	2.58(4)	3.442(3)	149(3)
N13-H13C•••O2	0.89(4)	2.18(4)	2.926(3)	142(3)
N13-H13C•••N11	0.89(4)	2.57(4)	3.158(3)	125(3)
N13-H13C•••N11	0.89(4)	2.54(3)	2.985(3)	112(3)
N14-H14A•••N7	0.90(4)	2.47(4)	3.243(4)	145(3)
N14-H14B•••N10	0.84(4)	2.33(4)	3.059(3)	147(3)
C2-H2•••N14	1	2.62	3.187(4)	116

3 Theoretical Study

Theoretical calculations were performed by using the Gaussian 09 (Revision E01) suite of programs. [1] The elementary geometric optimization and the frequency analysis were performed at the level of the Becke three parameter, Lee-Yan-Parr (B3LYP) functional with the 6-311+G** basis set. [2] All of the optimized structures were characterized to be local energy minima on the potential surface without any imaginary frequencies. Atomization energies were calculated by the G2. All the optimized structures were characterized to be true local energy minima on the potential-energy surface without imaginary frequencies.

The predictions of heat of formation (*HOF*) adopt the hybrid DFT-B3LYP methods with 6-311+G** basis set via designed isodesmic reactions. The isodesmic reaction processes, i.e., the number of each kind of formal bond is conserved, are used with application of the bond separation reaction (BSR) rules. The molecule is broken down into a set of two heavy-atom molecules containing the same component bonds. The isodesmic reactions used to derive the HOF of the title compounds are in Scheme S1. The change of enthalpy for the reactions at 298 K can be expressed as

$$\Delta H_{298} = \sum \Delta_f H_P - \sum \Delta_f H_R \qquad (1)$$

Where $\sum\Delta_f H_P$ and $\sum\Delta_f H_R$ are the *HOF* of reactants and products at 298 K, respectively, and ΔH_{298} can be calculated using the following expression:

$$\Delta H_{298} = \Delta E_{298} + \Delta(PV) = \Delta E_0 + \Delta ZPE + \Delta H_T + \Delta nRT \qquad (2)$$

Where ΔE_0 is the change in total energy between the products and the reactants at 0 K; ΔZPE is the difference between the zero-point energies (*ZPE*) of the products and the reactants at 0 K; ΔH_T is thermal correction from 0 to 298 K. The $\Delta(PV)$ value in eq (2) is the *PV* work term. It equals $\Delta(nRT)$ for the reactions of ideal gas. For the isodesmic reaction, $\Delta n = 0$, so $\Delta(PV) = 0$. On the left side of Eq. (1), apart from target compound, all the others are called reference compounds. The HOF of reference compounds is available from the experiments.

For energetic salts, the solid-phase heats of formation are calculated based on a Born-Haber energy cycle. The heat of formation of a salt can be simplified by the formula given in Equation (1):

$$\Delta H_f^0(\text{salt},298\text{ K}) = \Delta H_f^0(\text{cation},298\text{ K}) + \Delta H_f^0(\text{anion},298\text{ K}) - \Delta H_L \quad (1)$$

Where ΔH_L is the lattice energy of the salts, which could be predicted by using the formula suggested by Jenkins et al. [3] [Eq. (2)]

$$\Delta H_L = U_{POT} + [p(n_M/2 - 2) + q(n_X/2 - 2)]RT \quad (2)$$

where n_M and n_X depend on the nature of the ions, Mp^+ and Xq^-, and are equal to 3 for monatomic ions, 5 for linear polyatomic ions, and 6 for nonlinear polyatomic ions.The equation for lattice potential energy U_{POT} [Eq. (3)] has the form:

$$U_{POT}\,(\text{kJ}\cdot\text{mol}^{-1}) = \gamma\,(\rho_m/M_m)^{1/3} + \delta \quad (3)$$

Where ρ_m/g cm^{-3} is the density, M_m is the chemical formula mass of the ionic material, and values for the coefficients γ/kJ mol^{-1} cm and δ/kJ mol^{-1} are taken from the literature. [4,5]

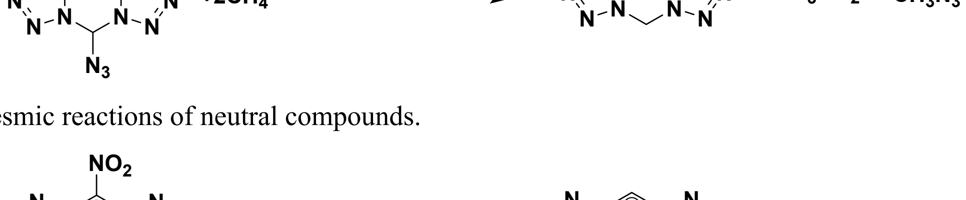

Scheme S1. Isodesmic reactions of neutral compounds.

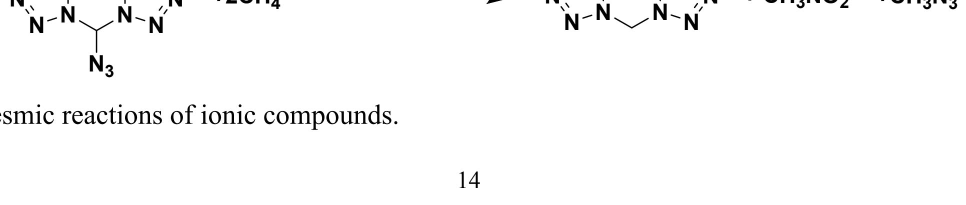

Scheme S2. Isodesmic reactions of ionic compounds.

Table S10 Ab initio computational values of small molecules used in isodesmic and tautomeric reactions.

Compound	E_0[a]	ZPE[b]	H_T[c]	HOF[d]
CH_4	-40.53	112.26	10.04	-74.6
CH_3N_3	-204.15	126.22	14.41	302
CH_3NO_2	-245.09	124.93	11.6	-80.8
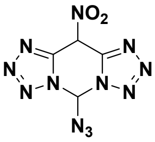	-960.68	307.55	2.48	890.4
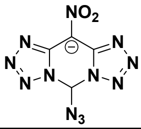	-960.27	264.04	2.48	924.73

[a]Total energy calculated by B3LYP/6-311+G**method (a.u);

[b]Zero-point correction (kJ mol^{-1});

[c] Thermal correction to enthalpy (kJ mol^{-1})

[d] Heat of formation (kJ mol^{-1}).

4.Reference

[1] Frisch, M. J.; Trucks, G. W.; Schlegel, H. B.; Daniels, A. D.; Farkas, O.; Foresman, J. B.; Ortiz, J. V.; Cioslowski, J.; Fox, D. J. Gaussian 09, Revision ABCD. 2009, 0123, Gaussian. Inc. Wallingford CT.

[2] Hariharan, P. C.; Pople, J. A.; Influence Of Polarization Functions On MO Hydrogenation Energies. Theor. Chim. Acta. 1973, 28, 213-222.

[3] Ochterski, J. W.; Petersson, G. A.; A Complete Basis Set Model Chemistry. V. Extensions to Six or More Heavy Atoms. Montgomery JA, J. Chem. Phys. 1996, 104, 2598-2619.

[4] Jenkins, H. D.; Tudeal, D.; Glasser, L. Lattice Potential Energy Estimation for Complex Ionic Salts from Density Measurements. Inorg. Chem. 2002, 41, 2364-2367.

[5] Jenkins, H. D.; Roobottom, H. K.; Passmore J, Glasser L. Relationships among Ionic Lattice Energies, Molecular (Formula Unit) Volumes, And Thermochemical Radii. Inorg. Chem. 1999, 38, 3609-3620.

5. NMR Spectras of Compounds 3-7 and DTAT-K

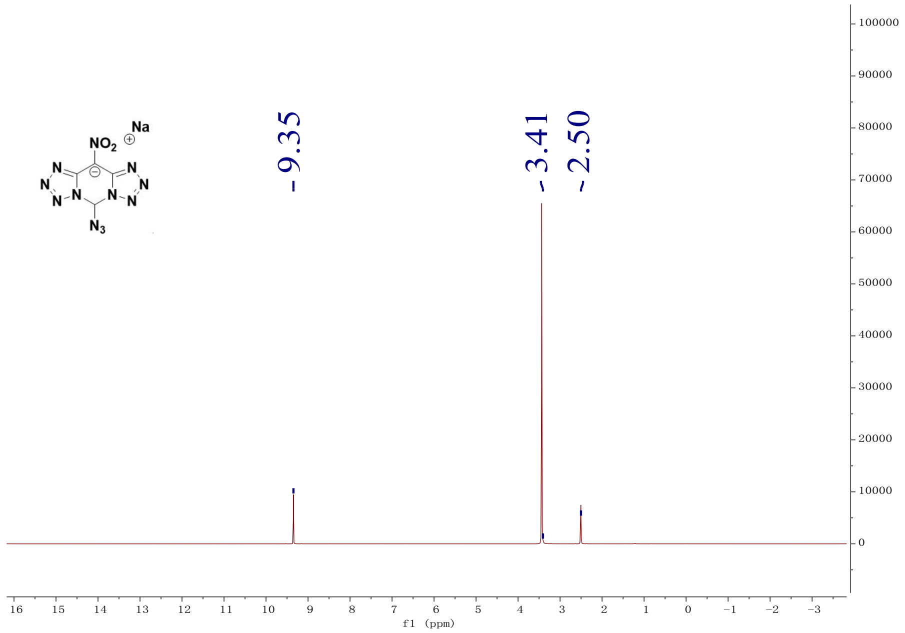

Figure S6 ^{1}H NMR spectra (500 MHz) of **3** in [D$_6$] DMSO at 25 ^{o}C.

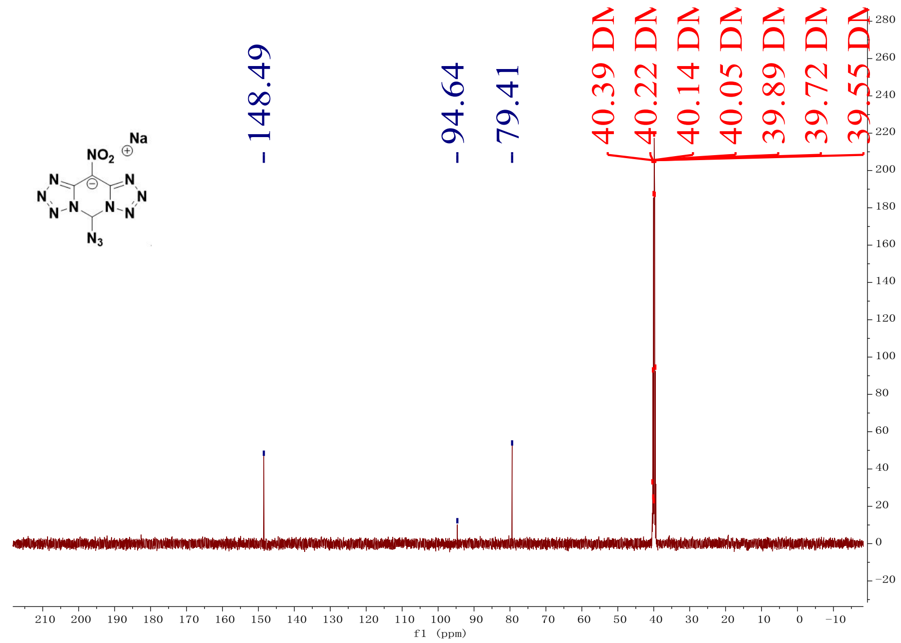

Figure S7 ^{13}C NMR spectra (125 MHz) of **3** in [D$_6$] DMSO at 25 ^{o}C.

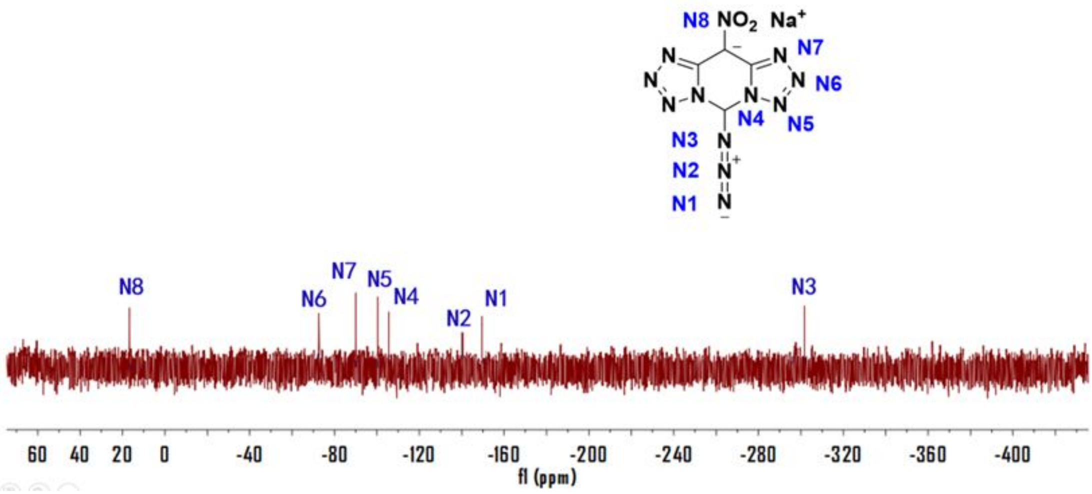

Figure S8 ^{15}N NMR spectra (50.7 MHz) of **3** in [D6] DMSO at 25 ^{o}C

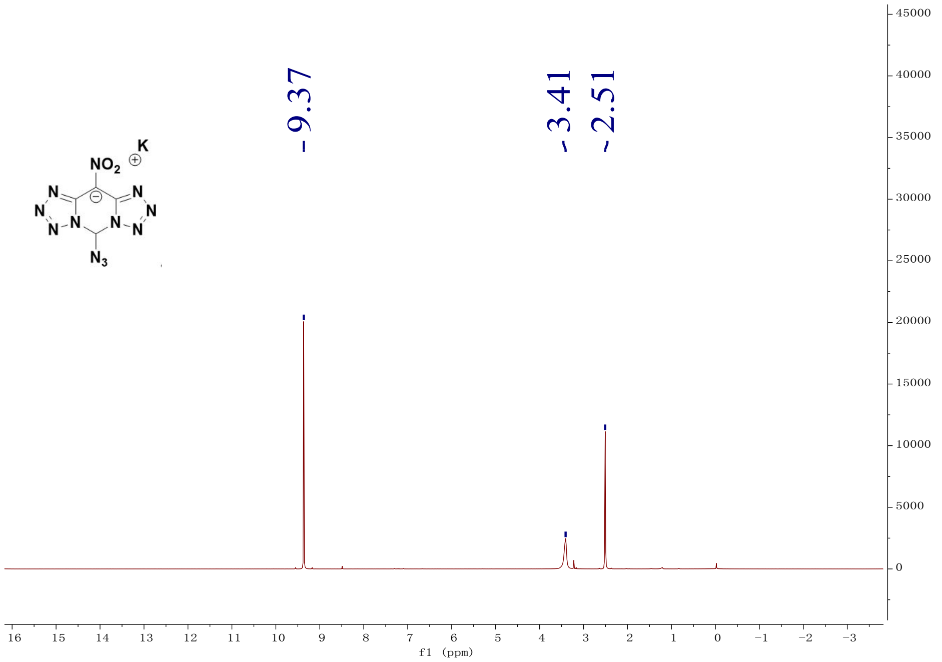

Figure S9 ^{1}H NMR spectra (500 MHz) of **DTAT-K** in [D_6] DMSO at 25 ^{o}C.

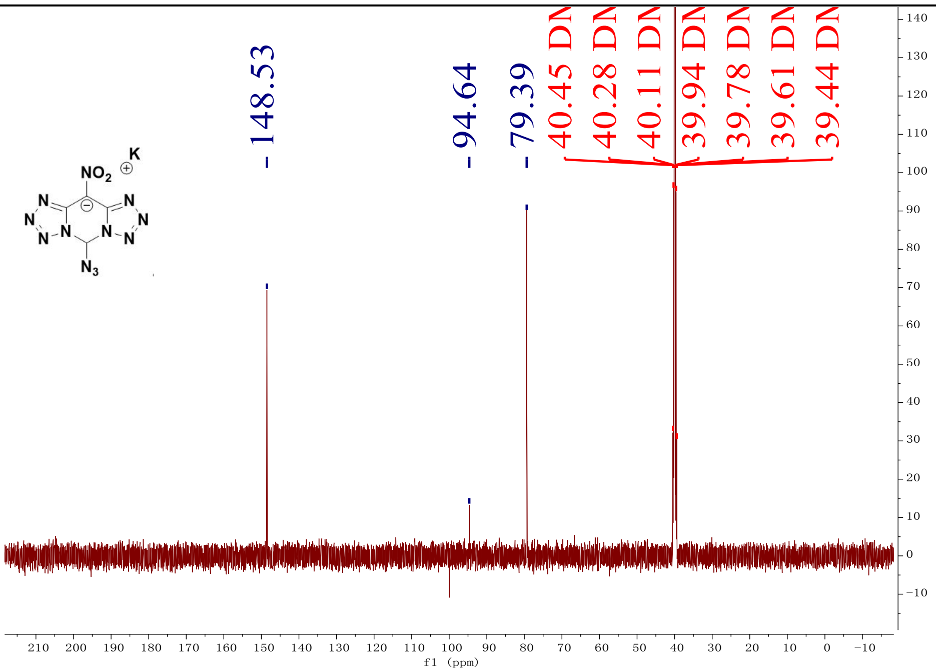

Figure S10 ^{13}C NMR spectra (125 MHz) of **DTAT-K** in $[D_6]$ DMSO at 25 °C.

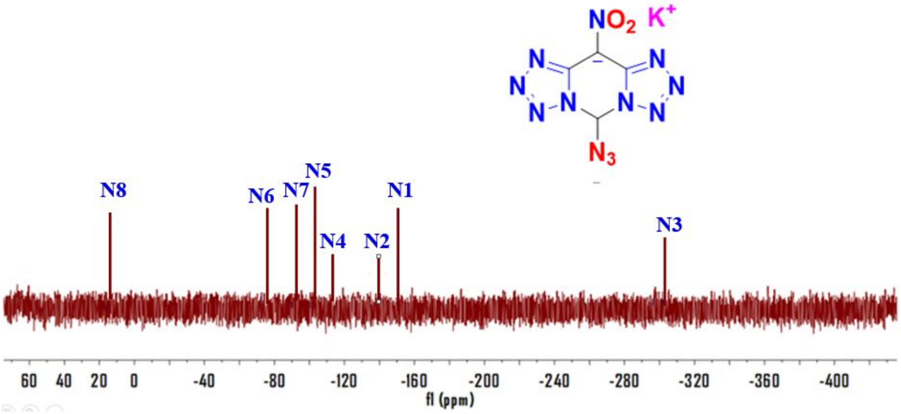

Figure S11 ^{15}N NMR spectra (50.7 MHz) of **DADT-K** in [D6] DMSO at 25 °C

`

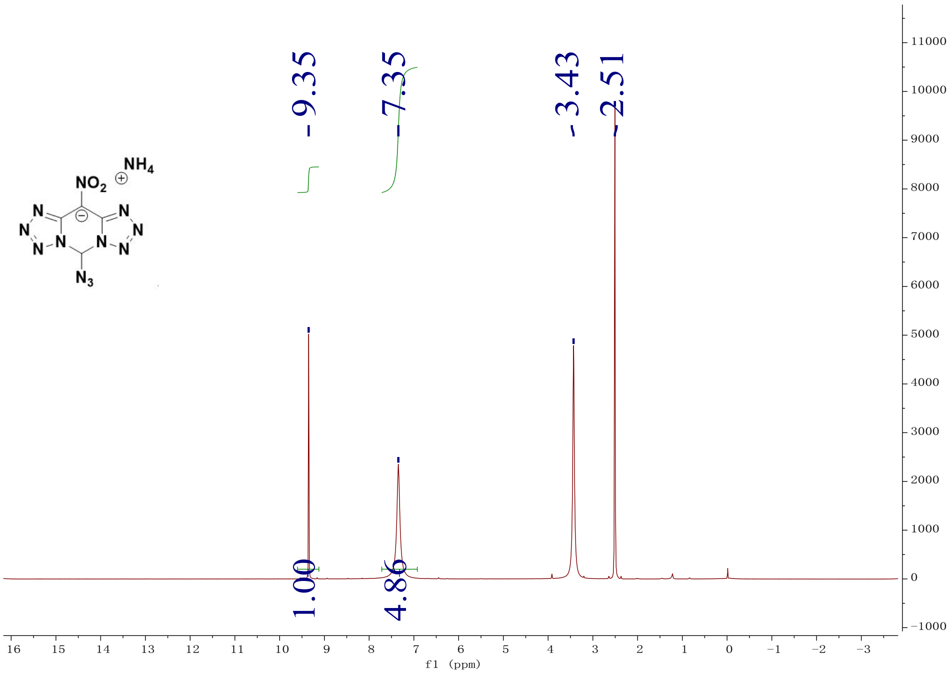

Figure S12 ^{1}H NMR spectra (500 MHz) of **5** in [D$_6$] DMSO at 25 ^{o}C.

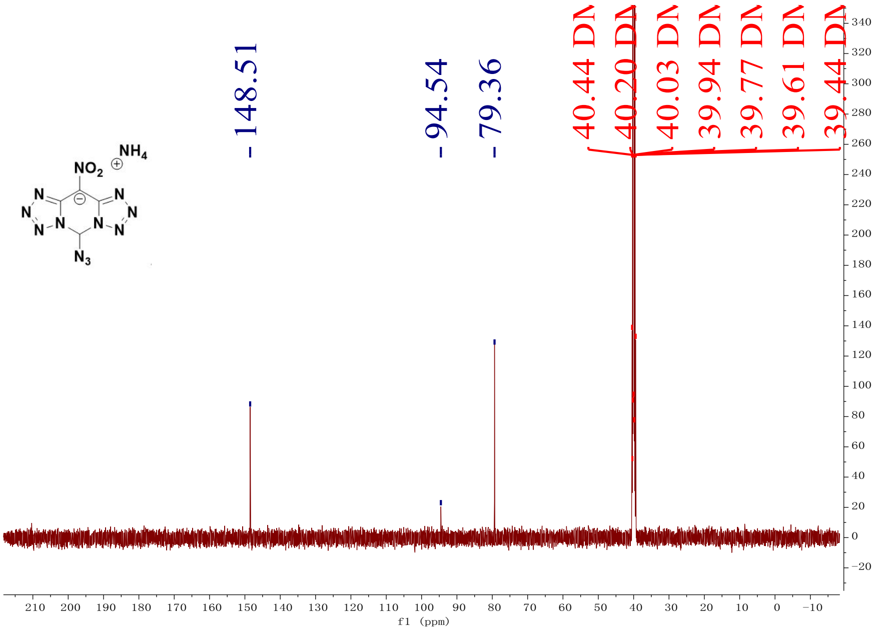

Figure S13 ^{13}C NMR spectra (125 MHz) of **5** in [D$_6$] DMSO at 25 ^{o}C.

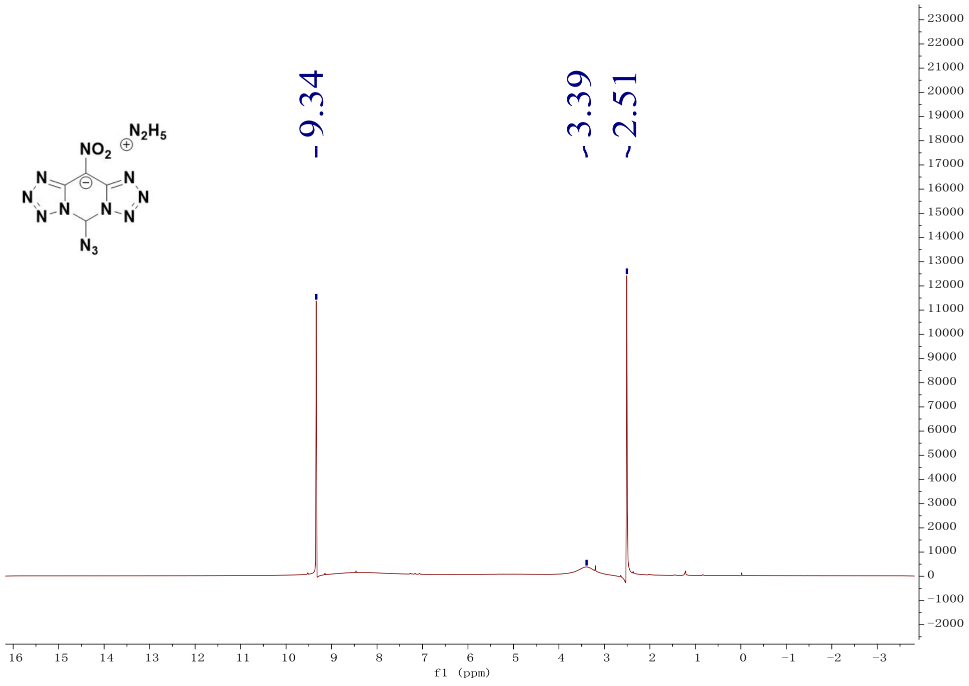

Figure S14 ^{1}H NMR spectra (500 MHz) of **6** in [D$_6$] DMSO at 25 ^{o}C.

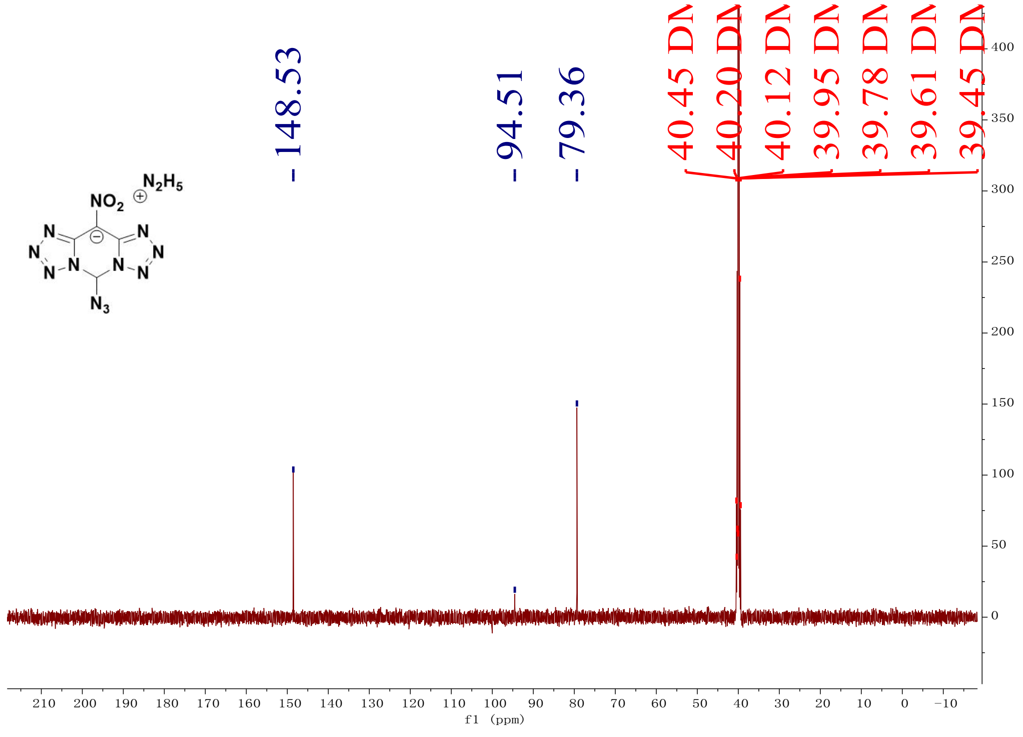

Figure S15 ^{13}C NMR spectra (125 MHz) of **6** in [D$_6$] DMSO at 25 ^{o}C.

`

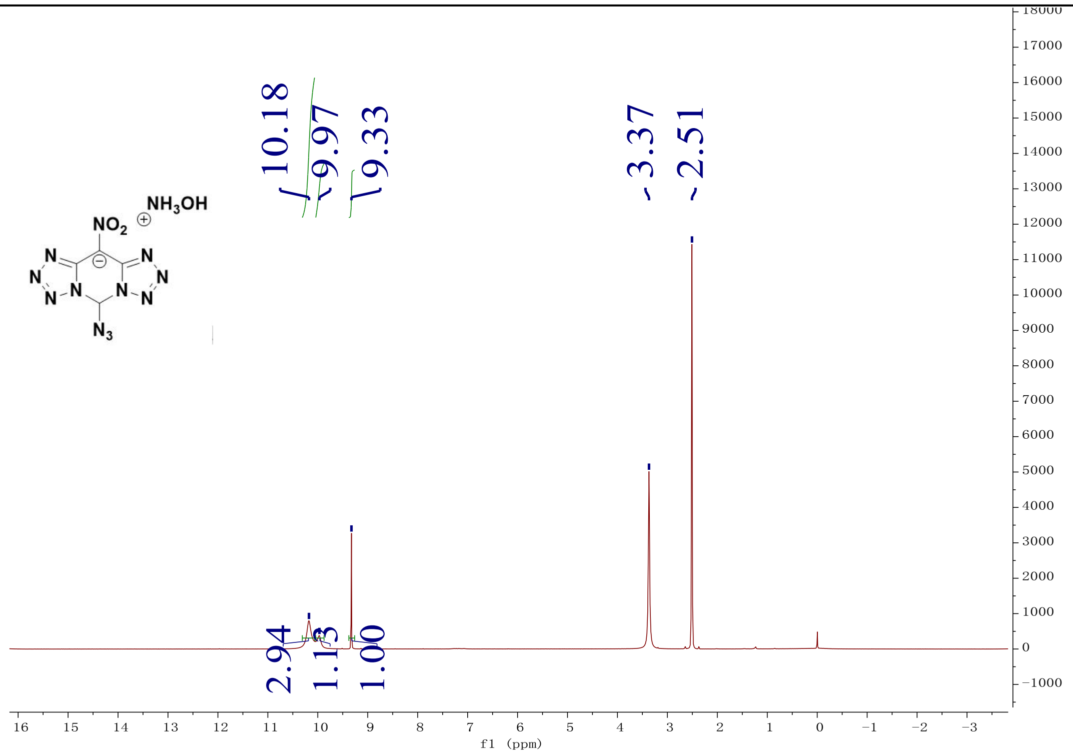

Figure S16 ^{1}H NMR spectra (500 MHz) of **7** in $[D_6]$ DMSO at 25 ^{o}C.

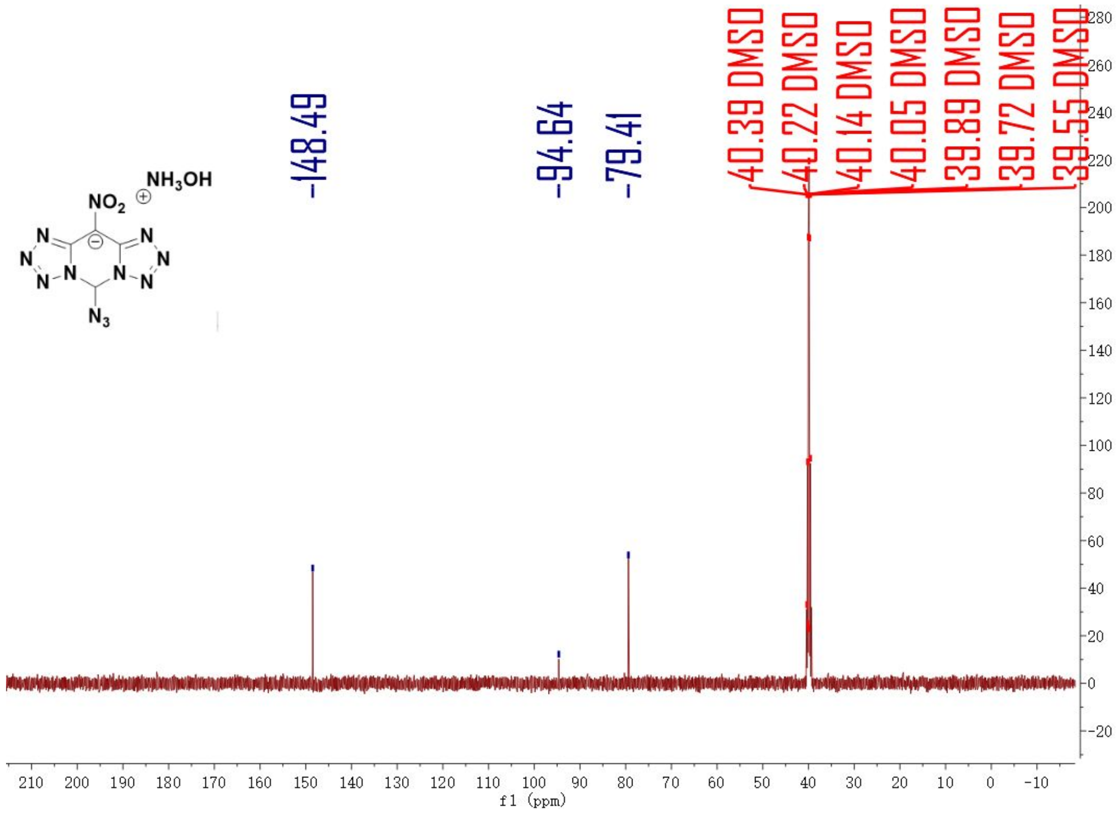

Figure S17 ^{13}C NMR spectra (125 MHz) of **7** in $[D_6]$ DMSO at 25 ^{o}C.

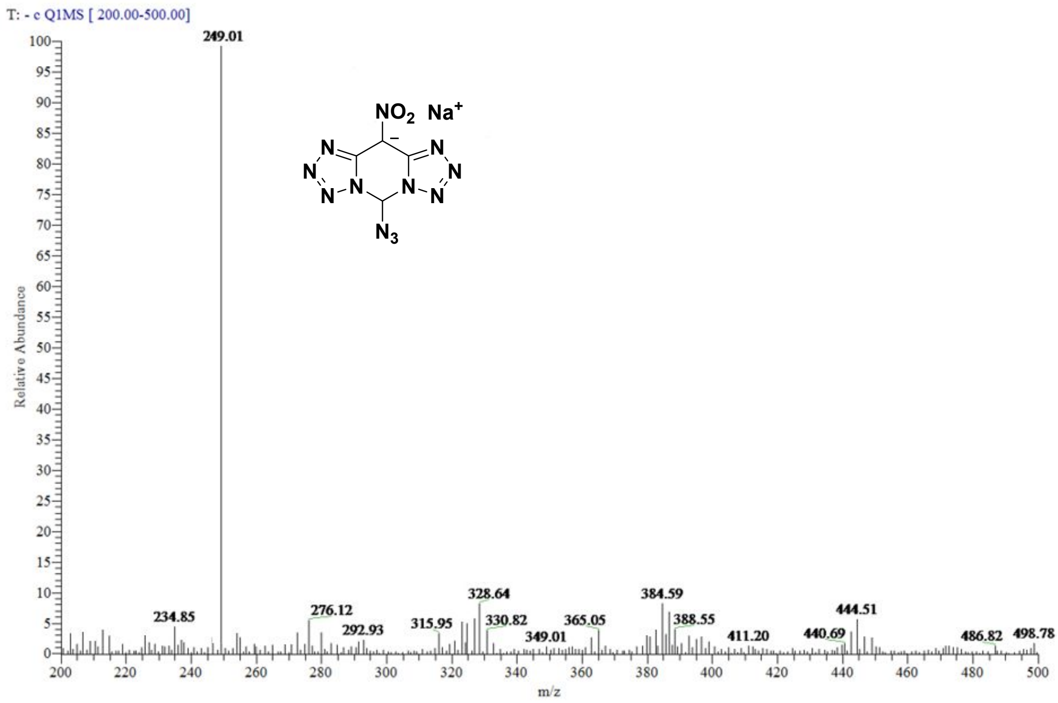

Figure S18 Mass spectra of compound **3**

6. The TG-DSC Curves of 4-6 and DTAT-K

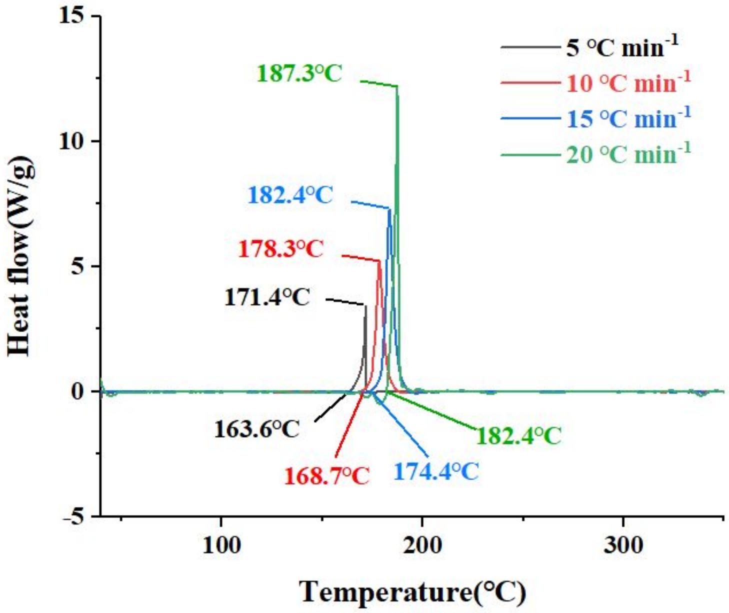

Figure S19 The DSC curves of **DTAT-K** with the heating rates of 5, 10, 15 and 20 ℃ min^{-1}

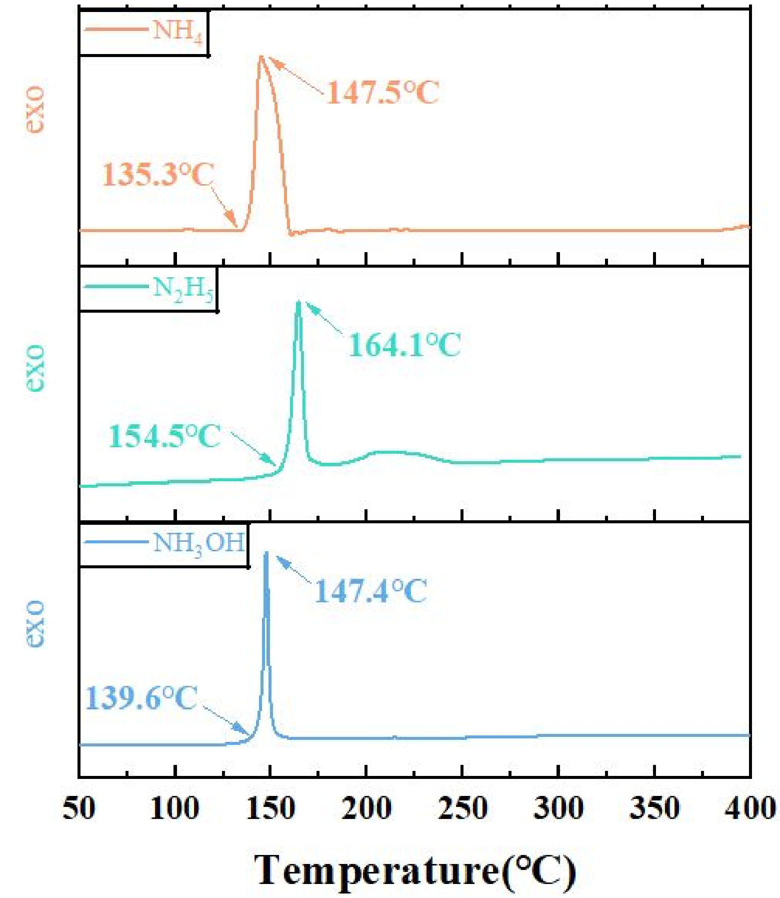

Figure S20 The TG-DSC curves of **4-6**.

7. The IR curves of 4-6 and DTAT-K

`

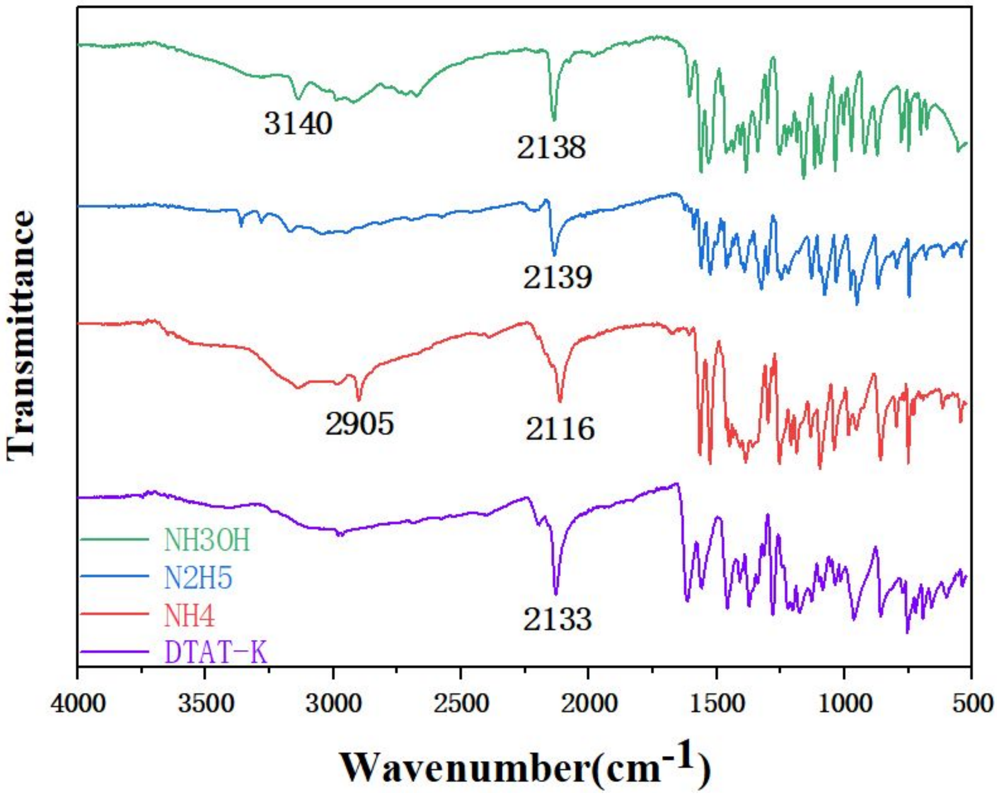

Figure S21 The IR curves of 4-6 and DTAT-K

`
